# SUMO-Interacting Motifs of Human TRIM5α are Important for Antiviral Activity

**DOI:** 10.1371/journal.ppat.1002019

**Published:** 2011-04-07

**Authors:** Gloria Arriagada, Lucia N. Muntean, Stephen P. Goff

**Affiliations:** 1 Department of Biochemistry and Molecular Biophysics, Columbia University, New York, New York, United States of America; 2 Howard Hughes Medical Institute, College of Physicians and Surgeons, Columbia University, New York, New York, United States of America; 3 Department of Microbiology and Immunology, Columbia University, New York, New York, United States of America; Fred Hutchinson Cancer Research Center, United States of America

## Abstract

Human TRIM5α potently restricts particular strains of murine leukemia viruses (the so-called N-tropic strains) but not others (the B- or NB-tropic strains) during early stages of infection. We show that overexpression of SUMO-1 in human 293T cells, but not in mouse MDTF cells, profoundly blocks N-MLV infection. This block is dependent on the tropism of the incoming virus, as neither B-, NB-, nor the mutant R110E of N-MLV CA (a B-tropic switch) are affected by SUMO-1 overexpression. The block occurred prior to reverse transcription and could be abrogated by large amounts of restricted virus. Knockdown of TRIM5α in 293T SUMO-1-overexpressing cells resulted in ablation of the SUMO-1 antiviral effects, and this loss of restriction could be restored by expression of a human TRIM5α shRNA-resistant plasmid. Amino acid sequence analysis of human TRIM5α revealed a consensus SUMO conjugation site at the N-terminus and three putative SUMO interacting motifs (SIMs) in the B30.2 domain. Mutations of the TRIM5α consensus SUMO conjugation site did not affect the antiviral activity of TRIM5α in any of the cell types tested. Mutation of the SIM consensus sequences, however, abolished TRIM5α antiviral activity against N-MLV. Mutation of lysines at a potential site of SUMOylation in the CA region of the Gag gene reduced the SUMO-1 block and the TRIM5α restriction of N-MLV. Our data suggest a novel aspect of TRIM5α-mediated restriction, in which the presence of intact SIMs in TRIM5α, and also the SUMO conjugation of CA, are required for restriction. We propose that at least a portion of the antiviral activity of TRIM5α is mediated through the binding of its SIMs to SUMO-conjugated CA.

## Author Summary

TRIM5α is an intrinsic immunity protein that provides a post-entry block of retroviral infection, which depends on its specific ability to recognize retroviral capsid (CA). Human TRIM5α is able to recognize and block infection by N-tropic murine leukemia virus (N-MLV) as well as other viruses. The exact mechanism by which TRIM5α exerts its action is still controversial. In this study, we have identified a new aspect of TRIM5α-mediated restriction of N-MLV: the involvement of the SUMO conjugation machinery. SUMO conjugation of MLV CA is an important step during viral infection, and our data suggest that innate immunity takes advantage of this process to restrict viral infection. We show that human TRIM5α protein contains two SUMO interacting motifs (SIMs) that are required for its antiviral activity against N-MLV. We propose that at least a portion of the antiviral activity of TRIM5α is mediated through the binding of its SIMs to SUMO-conjugated CA.

## Introduction

Cells have developed many mechanisms to restrict viral infection. The adaptative immune response provides major protection against viral pathogens, but recently dominant-acting inhibitory gene products, called restriction factors, have been discovered that also play an important role in limiting host susceptibility to viral infections. One class of such restriction factors blocks retroviral infection by targeting the incoming capsid protein (CA) (for review see [1]).

Early studies identified the Friend virus susceptibility factor 1 (*Fv1*) locus as a host gene determining the susceptibility of mice to infection by various strains of murine leukemia viruses (MLV) [2]. Two major alleles of *Fv1* were identified: *Fv1^n^* and *Fv1^b^*. *Fv1^n^* renders NIH/Swiss mice resistant to B-tropic MLV (B-MLV) infection and *Fv1^b^* renders BALB/c mice resistant to N-tropic virus [3], [4]. The critical difference between the N- and B-tropic MLVs was traced to specific residues of the viral capsid (CA) protein [5]–[7]. Some strains of MLV, including Moloney MLV, termed NB-tropic, are insensitive to Fv1 restriction [4]. The restriction by Fv1 occurs early in infection, after reverse transcription but before viral DNA integration, and is saturable by large amount of virus [8]–[10]. The mechanism by which Fv1 restricts MLV infection is unknown, but it is generally presumed that Fv1 somehow recognizes the incoming CA protein structure and prevents normal infection.

More recently, rhesus monkey TRIM5α and human TRIM5α were identified as intracellular restriction factors capable of blocking infection by human immunodeficiency virus type-1 (HIV-1) and N-MLV, respectively [11]–[15]. TRIM5α blocks retroviral replication early in the life cycle, after viral entry but before reverse transcription [16]–[21]. The same residues of MLV capsid determine sensitivity to the Fv1 and human TRIM5α-mediated restriction [14], [21]. Some human cell lines are able to potently block N-MLV infection (HeLa, TE671) while others (293T cells) do not block N-MLV or do so only weakly [20]. The mechanism by which TRIM5α restricts infection is unclear.

TRIM5α is a member of the tripartite motif family of proteins, characterized as having three domains: a RING domain, either one or two B-boxes, and a coiled-coil domain [22]. The C-terminus of TRIM5α, unlike that of most TRIMs, consists of a B30.2 domain. This domain binds to CA molecules of incoming retroviruses, and its sequence determines which retroviruses a specific TRIM5α will restrict [12], [14], [21], [23]–[27]. The RING domain is a cysteine-rich zinc binding domain commonly found in E3 ubiquitin ligases, and there is some evidence suggesting that TRIM5α could be a ubiquitin ligase [28]. The B-box domains are thought to act as protein-protein interaction domains and thereby determine RING box ubiquitin ligase substrate specificity [29]. The coiled-coil domain has been shown to be involved in homo- and hetero-multimerization of the TRIM proteins, and deletion of this domain in TRIM5α completely abrogates HIV-1 and N-MLV restriction [30]–[32].

SUMO proteins are small ubiquitin-related proteins that become conjugated to cellular substrates and regulate diverse cellular processes, including intracellular trafficking, cell cycle progression, transcription, DNA repair and nuclear receptor activities (for review see [33], [34]). SUMO conjugation, like ubiquitination, requires an E1 activating enzyme (in the case of SUMO, these are AOS1-UBA2), an E2 conjugation enzyme (UBC9) [35], [36] and often an E3 ligase (RanBP2 and PIAS 1, -3 and -4/y) which recognize the substrate and determine target specificity [37]–[39]. In mammals, three SUMO paralogues are commonly expressed: SUMO-1, SUMO-2 and SUMO-3. SUMO-1 is distinct from SUMO-2 and SUMO-3; SUMO-2 and SUMO-3 are 97% identical to each other but only 47% identical to SUMO-1. SUMO-1 and SUMO-2/3 are conjugated to different target proteins *in vivo* [40], [41], [42] and likely serve distinct functions. SUMO proteins are usually transferred to lysines of a UBC9 binding site motif of consensus sequence ΨKxE (where Ψ is a hydrophobic residue and x any amino acid) [43], [44], though lysines in other contexts can be modified.

In addition to targeting different substrate proteins, the functional properties of SUMO isoforms might also reflect their ability to mediate distinct protein-protein interactions *in vivo*. Recent work has identified specific motifs that mediate non-covalent interactions with SUMO modified proteins [43], [45], [46]. The best characterized of the SUMO-interacting motifs (SIMs) have the consensus sequence V/I/L-x-V/I/L-V/I/L or V/I/L-V/I/L-x-V/I/L (where x is any amino acid) [46], [47].

In the past few years, there have been many reports demonstrating the involvement of SUMO conjugation in virus replication. In some instances, conjugation of SUMO to either viral proteins or host proteins can impair viral infection, and hence, viruses have found ways to interfere with the pathway [48]–[50]. In other instances, SUMO conjugation of viral proteins can be essential for viral replication [41], [48], [51]. It has been reported that Gag proteins from Mazon-Pfizer monkey virus, Moloney murine leukemia virus, and HIV-1 interact with the SUMO conjugation pathway [52]–[54]. Our laboratory has previously reported that the E2 and E3 SUMO-conjugating enzymes, UBC9 and PIAS4/y, interact with the capsid (CA) protein of Moloney murine leukemia virus (MoMLV or NB-tropic MLV). The UBC9 and PIAS4/y binding sites within CA were identified, and it was also demonstrated that co-expression of CA and tagged-SUMO-1 proteins resulted in SUMO conjugation of CA *in vivo*. Mutation of lysine residues to arginine near the UBC9 binding site and ablation of the PIAS4/y binding site reduced or eliminated CA SUMO conjugation, and impaired virus replication. This block occurred in the early stages of viral infection, after reverse transcription and before nuclear entry and viral DNA integration. The findings suggest a role for the SUMO machinery in the early stages of viral infection [54].

In an effort to further elucidate the relationship between the SUMO conjugation pathway and early events of the MLV life cycle, we tested the effects of manipulating the components of the SUMO transfer machinery in several cell lines. Surprisingly, we found that the normally weak N-MLV restriction by TRIM5α in human cells is profoundly enhanced by overexpression of SUMO-1. The presence of two SIMs in TRIM5α is required for the enhanced N-MLV restriction. Mutation of lysines preventing CA SUMOylation, abolish TRIM5α-mediated restriction of N-MLV. Our data suggest a novel aspect of the TRIM5α-mediated restriction of N-tropic MLV, in which the presences of intact SIMs in TRIM5α, and SUMO conjugation of CA, are required for N-MLV restriction. We propose that TRIM5α recognition of CA is augmented by binding SUMO-modified CA via its SUMO-interacting motif.

## Results

### Overexpression of human SUMO-1 enhances the block of N-tropic MLV infection in 293T cells

**Figure 1:**
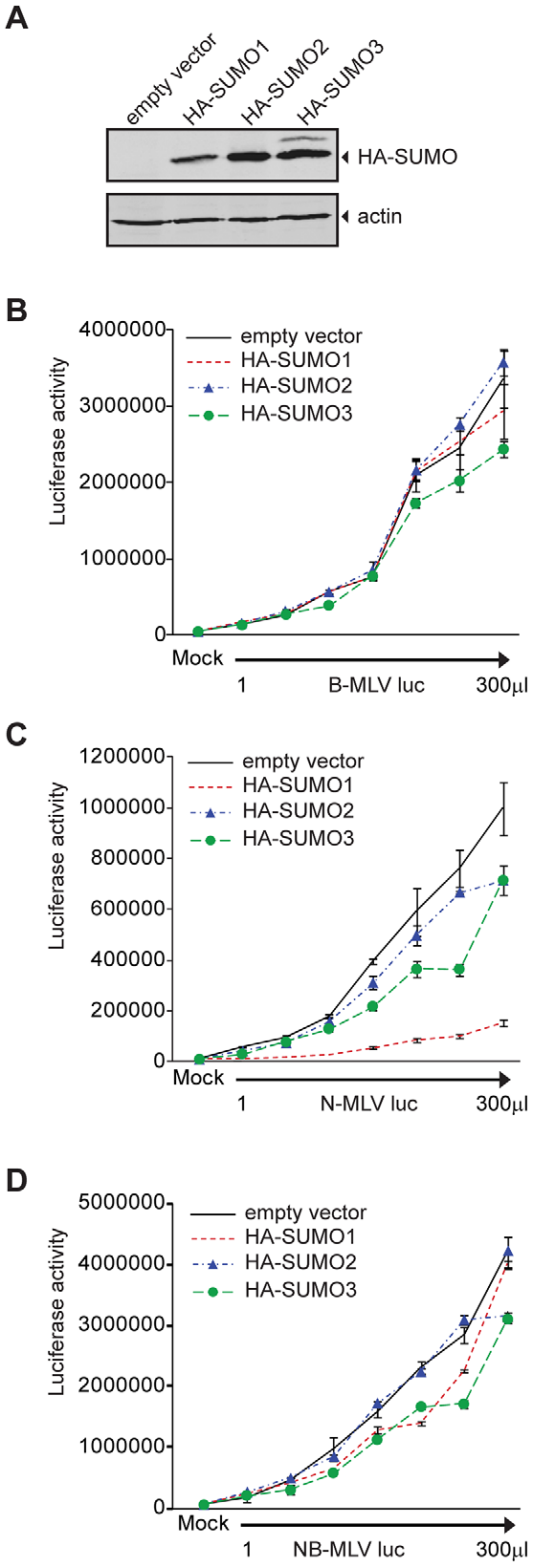
Overexpression of SUMO-1 blocks N-tropic MLV infection in 293T cells. **A**. 293T cells stably expressing an empty vector, HA-SUMO-1, HA-SUMO-2 or HA-SUMO-3 were generated. The presence of the overexpressed HA-tagged SUMO paralogs was detected by Western blot using an antibody directed against the HA-tag (upper panel), using actin as a control (bottom panel). **B–D**. The 293T empty vector control cell line or HA-SUMO-1, HA-SUMO-2 or HA-SUMO-3 overexpressing cell lines were mock treated or infected with increasing amount of VSV-G pseudotyped B-MLV luc (**B**), N-MLV luc (**C**) or NB-MLV luc (**D**). Forty-eight hours after infection, luciferase activity was measured. One representative experiment of five independent experiments is shown. Error bars indicate standard deviation among triplicates in the same experiment.

To explore the significance of SUMO conjugation in the early stages of viral infection, we transduced 293T cells with an empty retroviral vector or a vector encoding HA-tagged versions of human SUMO-1, SUMO-2 or SUMO-3. Pools of cell lines stably expressing these proteins were selected. The presence of the HA-tagged SUMO proteins was detected by Western blot (Figure 1A). The empty vector control, HA-SUMO-1, HA-SUMO-2, and HA-SUMO-3 overexpressing cell lines were then assayed in single round infection experiments. The different cell lines were infected in parallel with increasing amounts of VSV-G pseudotyped B- (Figure 1B), N- (Figure 1C), or NB-tropic MLV (Figure 1D) virus particles delivering a firefly luciferase reporter (B-MLV luc, N-MLV luc and NB-MLV luc respectively). The overexpression of HA-SUMO-1 in 293T cells reduced the infectivity of N-MLV by an average of more than 8-fold as compared to the empty vector cell line (Figure 1C). In contrast, SUMO-1 overexpression had no significant effect on susceptibility to infection by B-MLV (Figure 1B) or NB-MLV (Figure 1D). Overexpression of HA-SUMO-2 did not affect susceptibility to infection by any of the viruses. Overexpression of HA-SUMO-3 reduced the susceptibility to infection by N-MLV by only 2 fold, again with no effect on infection by B- or NB-MLV. These data show that SUMO-1 overexpression induces or enhances a block to N-MLV infection in 293T cells.

**Figure 2:**
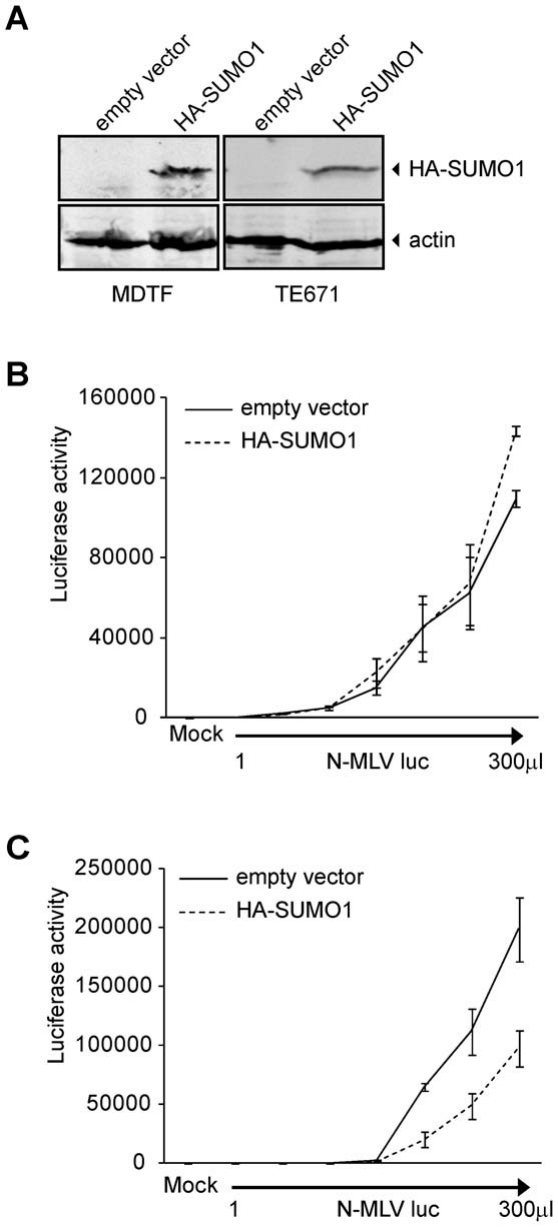
Overexpression of SUMO-1 in MDTF and TE671 cells. **A**. Stable MDTF and TE671 cells expressing an empty vector or HA-SUMO-1 were generated, and overexpressed HA-tagged SUMO-1 and actin were detected by Western blot. **B**. The MDTF empty vector control or HA-SUMO-1 over-expressing cell lines were mock treated or infected with increasing amounts of VSV-G pseudotyped N-MLV luc. **C**. The TE671 empty vector control cell line or HA-SUMO-1 overexpressing cell line were mock treated or infected with increasing amount of VSV-G pseudotyped N-MLV luc. Forty-eight hours after infection, luciferase activity was measured. One representative experiment of three independent experiments is shown. Error bars indicate standard deviation among triplicates in the same experiment.

This data raised the possibility that SUMO-1 overexpression could also block N-MLV infection in murine cells that are non-restrictive for N-MLV, such as the Fv1-null *Mus dunni* tail fibroblasts (MDTF). We transduced MDTF cells with the retroviral vectors encoding HA-SUMO-1 used above, and detected the presence of HA-SUMO-1 by Western blot (Figure 2A). Overexpression of SUMO-1 in MDTF did not affect N-MLV infectivity when compared to the empty vector cell line (Figure 2B). This result indicates that the inhibition of N-MLV infection by overexpression of SUMO-1 is cell-type dependent, and could require a baseline level of restriction.

293T cells have been described as non-restrictive or very weakly restrictive for N-MLV infection when compared to other human cell lines such as TE671 or HeLa [20]. To test if overexpression of SUMO-1 in other more restrictive human cells could enhance the restriction to N-MLV, we transduced TE671 cells with retroviral vectors encoding HA-SUMO-1 used above, and confirmed expression by Western blot (Figure 2A). Overexpression of SUMO-1 in TE671 cells reduced infection of N-MLV by an average of 3 fold as compared to the empty vector cell line (Figure 2C). This result suggests that overexpression of SUMO-1 can enhances a restriction activity present in multiple human cell lines.

Since overexpression of SUMO-1 is enhancing restriction in human cells, we wondered if the knock down of endogenous SUMO-1 would be able to release restriction. We knocked down the endogenous SUMO-1 in 293T, HeLa and TE671, then we infected the cells with N-MLV or B-MLV luc, virus infection was not significantly increased (data not shown). These results are very difficult to interpret; SUMO-1 is required for early events during infection [54] and any effect observed with the different shRNA used was equivalent for both N-MLV and B-MLV luc infection, although the restriction to N-MLV infection in HeLa and TE671 cells is still present.

### The SUMO-1-mediated block of N-MLV is dependent on CA residue 110

**Figure 3:**
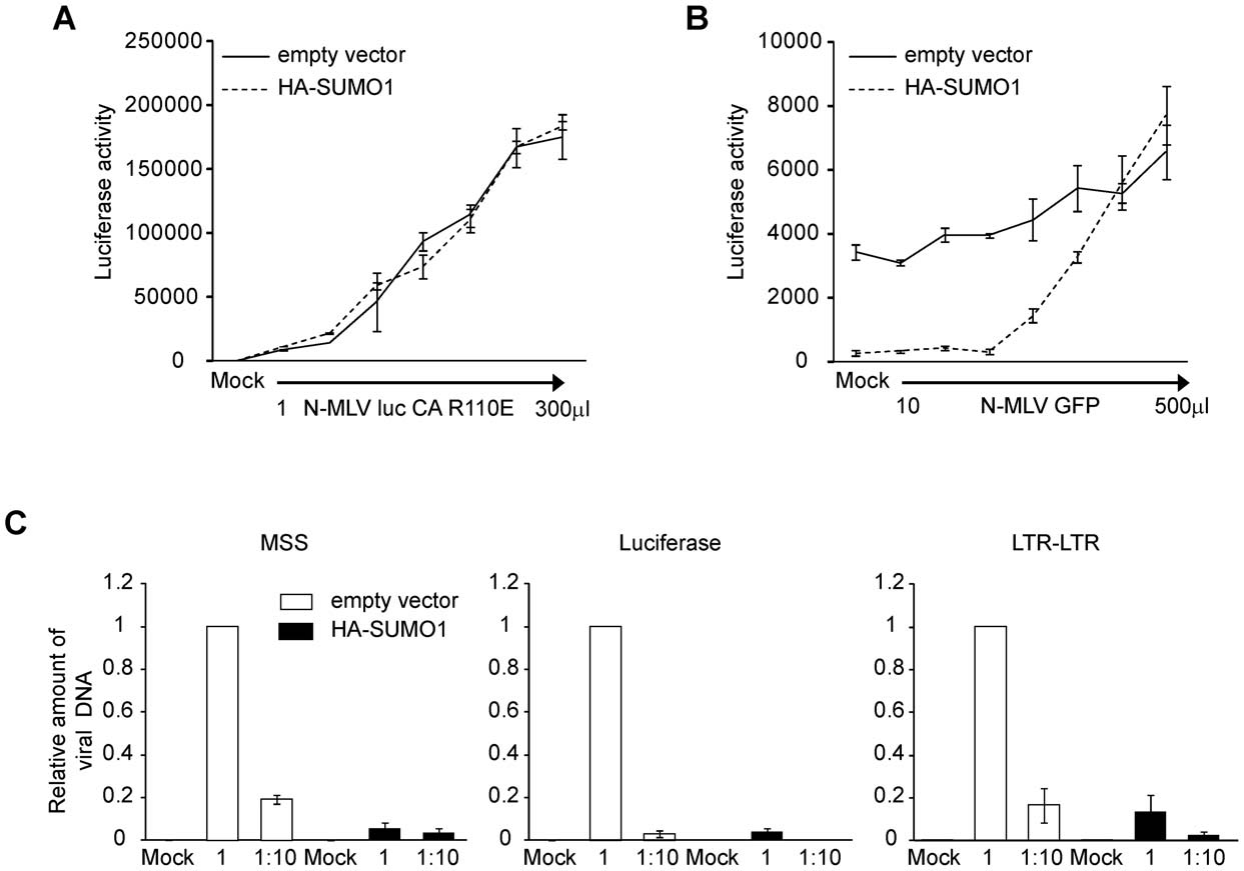
Characterization of SUMO-1 block of N-MLV infection in 293T cells. **A.** The 293T empty vector control or HA-SUMO-1 cell lines were infected with increasing amounts of a mutant version of N-MLV luc in which amino acid 110 of the CA protein was mutated from arginine to glutamic acid (R110E). Luciferase activity was measured forty-eight hours post-infection. One representative experiment of three independent experiments is shown. **B.** The 293T empty vector control or HA-SUMO-1 cell lines were infected with increasing amounts of VSV-G-pseudotyped N-MLV expressing a GFP reporter, and four hours post-infection, cells were superinfected with a fixed amount of VSV-G-pseudotyped N-MLV luc. Luciferase activity was measured forty-eight hours post-infection. One representative experiment of four independent experiments is shown. **C.** The 293T empty vector control or HA-SUMO-1 overexpressing cell lines were infected with undiluted (1∶1) or 10-fold diluted (1∶10) VSV-G-pseudotyped N-MLV luc. Mock treated cells were included as a negative control. Total DNA was isolated twenty hours after infection, and the amount of viral DNA synthesized in the infected cells was measured by quantitative PCR. Primers specific for the minus-strand strong stop (MSS) DNA (left panel), Luciferase gene (middle panel) or LTR-LTR junction (right panel) were used. The values were normalized to GAPDH DNA and expressed as fold over empty vector. Error bars indicate standard deviation among triplicates in the same experiment (**A and B**) or 3 different experiments (**C**).

Our data show that SUMO-1 specifically blocks N-tropic MLV and not B- or NB-tropic MLV. This suggests that the viral capsid, and its associated tropism, is a critical determinant of the antiviral effects of SUMO-1 on N-MLV. Capsid (CA) protein amino acid 110 appears to be the most important determinant of Fv-1 N (specified by R110) and B (specified by E110) tropisms [7]. To determine if CA is also the target for the block observed in SUMO-1-overexpressing 293T cells, we generated a mutant version of N-MLV luc in which amino acid 110 in CA was mutated from arginine to glutamic acid (N-MLV luc CA R110E), a change known to convert Fv-1 sensitivity from N to B-tropism. N-MLV luc CA R110E infection was not blocked by HA-SUMO-1 overexpression in 293T when compared with the empty vector cell line (Figure 3A). Therefore the antiviral effects of SUMO-1 on N-MLV are dependent on CA amino acid 110.

### The SUMO-1-mediated block of N-MLV can be abrogated by pretreatment with virus particles

The observation that the antiviral effects of SUMO-1 are CA-dependent and cell-type specific suggests that SUMO-1 overexpression enhances the antiviral activity of an intrinsic restriction factor. Human cells contain the restriction factor TRIM5α, which confers resistance to N-MLV but not B- or NB-MLV infection [13], [20], [32], [55]. TRIM5α antiviral activity was found to be dependent on CA amino acid 110 and could be abrogated with high multiplicities of infection [20], [56]. To determine whether the SUMO-1 enhanced block could also be abrogated, 293T cells transduced with the empty vector control or HA-SUMO-1 were pretreated with increasing amounts of N-MLV containing a green fluorescent protein (GFP) reporter gene (N-MLV GFP). Four hours later, the cells were superinfected with a fixed amount of N-MLV luc. Those 293T cells expressing the empty vector did not significantly restrict N-MLV luc infection (Figure 1C). Pretreatment of those cells with N-MLV GFP had little effect on the luciferase activity after infection with N-MLV luc (Figure 3B). Pretreatment of 293T cells overexpressing HA-SUMO-1 with N-MLV GFP at high doses, however, resulted in a dose-dependent loss of the SUMO-1-mediated block to N-MLV luc infection. Thus, as observed for the TRIM5α-mediated restriction, the SUMO-1 block of N-MLV infection can be abrogated.

### The SUMO-1 block of N-MLV occurs prior to reverse transcription

Under normal circumstances, TRIM5α blocks retroviral replication early in the life cycle, after viral entry but before reverse transcription [19], [57]. To determine whether the block is occurring at a similar time in the SUMO-1 overexpressing cells, the course of viral DNA synthesis was examined by qPCR after acute infection. 293T cells stably transduced with the empty vector or HA-SUMO-1 were infected with N-MLV luc at two different dilutions. The levels of amplified viral DNA products correlated well with the levels of input virus in the empty vector control cell line. Examination of an early step in reverse transcription, using primers that detect the minus strand strong stop (MSS) DNA (the first detectable product of viral DNA synthesis), revealed a significant reduction in MSS DNA products in the SUMO-1-overexpressing cell line as compared to the empty vector cell line (Figure 3C, left panel). In correlation with this, very low levels of luciferase DNA sequences, which correspond to linear fully reverse transcribed viral DNA from our luciferase reporter (Figure 3C, middle panel), and nuclear viral DNA forms, analyzed by detection of the 2-LTR junction (Figure 3C, right panel) were detected in the SUMO-1-overexpressing cell line as compared to the empty vector cell line. These data shows that SUMO-1 overexpression resulted in an early block in the viral life cycle, at a step prior to reverse transcription, and that the block to early forms continues to affect later DNA forms in the course of infection.

### The SUMO-1-mediated block of N-MLV is mediated by human TRIM5α

TRIM5α-mediated restriction of N-MLV infection is dependent on CA amino acid 110, occurs prior to reverse transcription, and can be saturated with high multiplicities of infection [14], [20], [56]. The SUMO-1 block of N-MLV infection in 293T has the same characteristics, suggesting that TRIM5α could be mediating the block of N-MLV by SUMO-1 overexpression.

**Figure 4:**
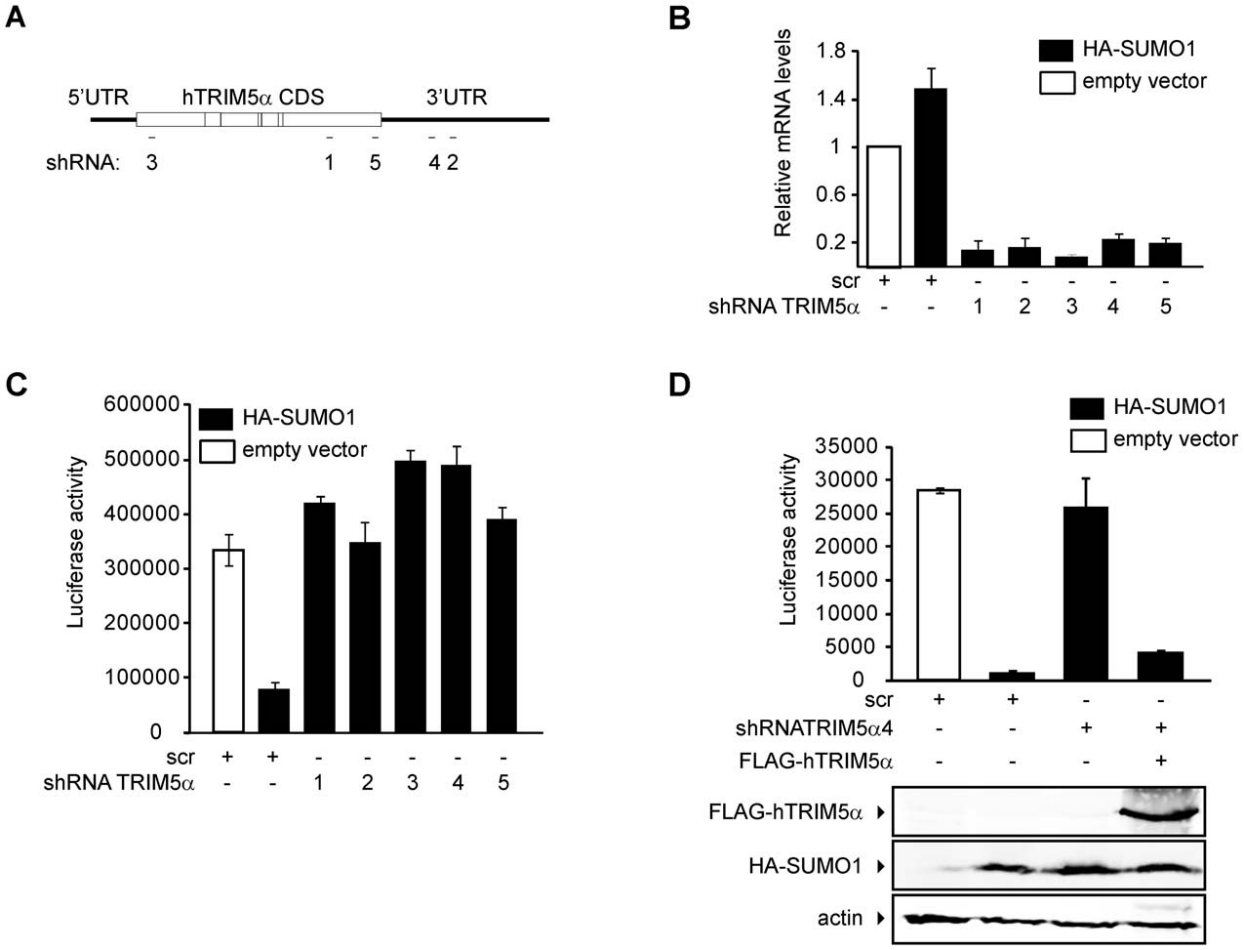
SUMO-1 block of N-MLV infection on 293T cells is mediated by TRIM5α. **A.** Schematic representation of the target site of five different shRNAs directed to the human TRIM5α messenger RNA. **B.** The 293T empty vector control cell line stably expressing a control scrambled (scr) shRNA (white bar) or the HA-SUMO-1 overexpressing cell line stably expressing a control scrambled shRNA, or different shRNAs directed to human TRIM5α mRNA (black bars), were generated. RNA was extracted, and the mRNA levels of TRIM5α were determined by quantitative PCR. The values were normalized to GAPDH mRNA and expressed as fold over empty vector-scr. **C.** The 293T expressing the control scr shRNA or the different shRNAs directed to TRIM5α mRNA were infected with a fixed multiplicity of N-MLV luc. Forty-eight hours after infection luciferase activity was measured. **D.** The 293T expressing the control scr shRNA or the shRNA4 were transiently transfected with an empty plasmid or a plasmid encoding human TRIM5α with a FLAG-tag as indicated. Twenty-four hours after transfection, cells were infected with a single dose of N-MLV luc. Forty-eight hours after infection, luciferase activity was measured, and Western blots were performed on the same extract to verify the expression of FLAG-TRIM5α using an anti-FLAG antibody (upper panel), an anti-HA antibody to verify the expression of HA-SUMO-1 (middle panel) and an anti-actin antibody as a control (bottom panel). Error bars indicate standard deviation among triplicates in the same experiment.

To determine whether TRIM5α mediates SUMO-1 restriction of N-MLV, we knocked down the expression of TRIM5α in the HA-SUMO-1 overexpressing cell line using shRNAs specific for the coding sequence or the 3′ UTR region of human TRIM5α mRNA (Figure 4A). An shRNA containing a scrambled, nonsilencing (scr) sequence was used as a control. TRIM5α mRNA was efficiently reduced by all of the targeted shRNAs tested, as determined by qPCR (Figure 4B). Expression of all five of the shRNAs directed specifically to the TRIM5α transcript abolished the HA-SUMO-1 block to N-MLV luc infection (Figure 4C), while expression of the control scr shRNA had no effect. This result strongly suggests that human TRIM5α is required for the SUMO-1 block of N-MLV infection.

To confirm that elimination of the SUMO-1 block to N-MLV infection by TRIM5α shRNA was due to reduced levels of TRIM5α and not the result of an off-target effect, we transiently transfected a plasmid encoding a FLAG-tagged human TRIM5α ORF, but lacking any TRIM5α UTR sequences into the HA-SUMO-1/shRNA4 cell line, in which the shRNA is directed to the 3′UTR region of the TRIM5α transcript. The SUMO-1 block to N-MLV was restored when we reintroduced the shRNA-resistant TRIM5α in the HA-SUMO-1/shRNA4 cell line (Figure 4D). We confirmed the restored TRIM5α expression by Western blotting of the same extracts used for the luciferase assay (Figure 4D bottom). These results demonstrate that the block of N-MLV infection upon SUMO-1 overexpression requires human TRIM5α.

**Figure 5:**
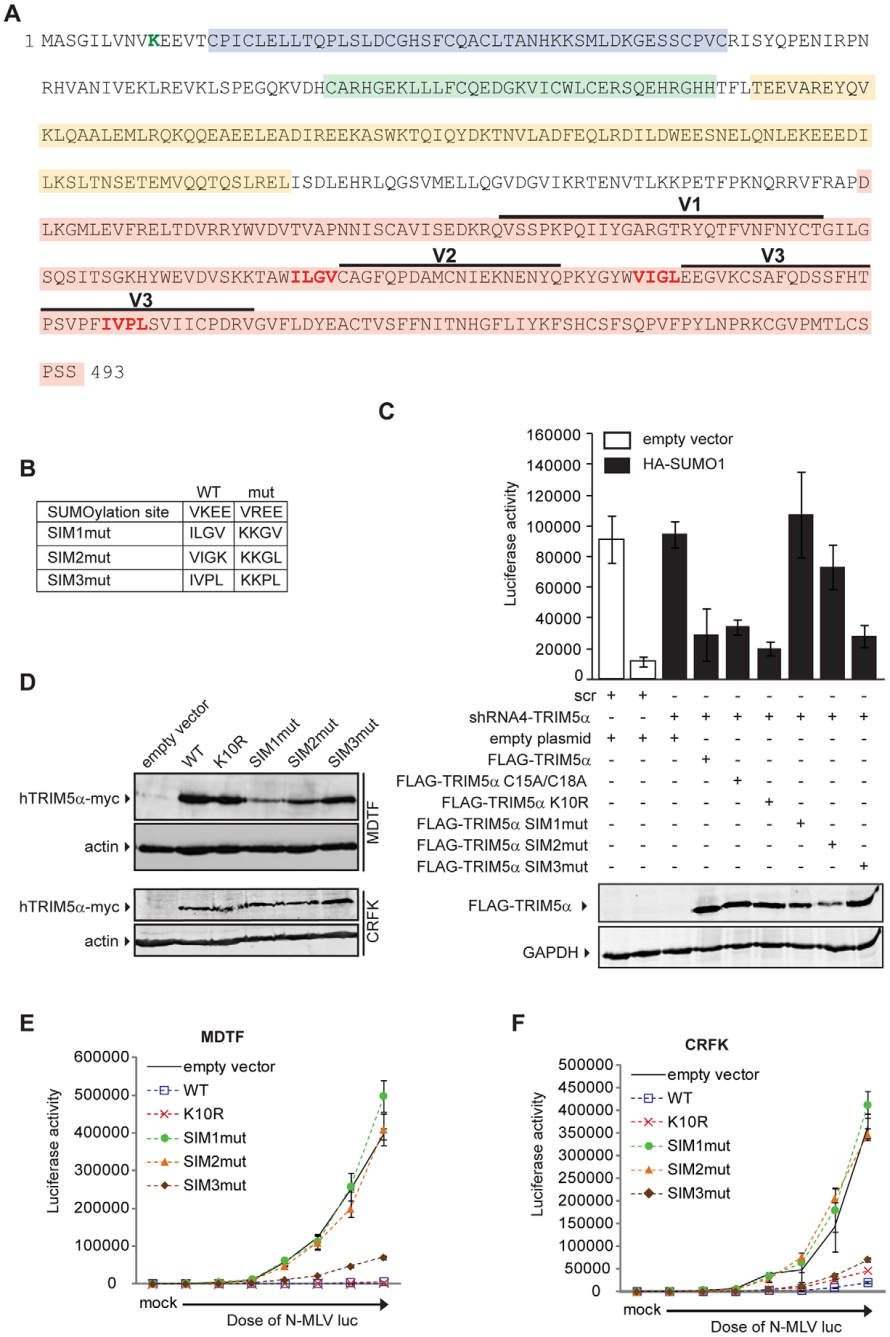
SUMO interacting motifs present in human TRIM5α are important for restriction of N-MLV. **A.** Amino acid sequence of human TRIM5α. The RING (blue), B-box (green), coiled-coil (yellow), B30.2 (pink) domains, and the location of a consensus SUMO conjugation site (green letter) and three putative SUMO interacting motifs (SIM) (red letters) are indicated. The variable regions of the B30.2 domain are depicted with horizontal bars above. **B.** The substitution mutations are indicated. WT: wild-type amino acid sequence; mut: mutated amino acid sequence. **C.** The 293T empty vector cell line expressing scr shRNA or the HA-SUMO-1 expressing scr shRNA were transfected with an empty plasmid, and the HA-SUMO-1 expressing shRNA4 (shRNA4-TRIM5α) cells were transiently transfected with an empty plasmid or plasmids encoding FLAG-tagged wild-type human TRIM5α or the C15A/C18A, K10R, SIM1mut, SIM2mut and SIM3mut mutants. Twenty-four hours after transfection, cells were infected with a single dose of N-MLV luc. Forty-eight hours after infection, luciferase activity was measured and Western blotting was performed using the same extract to verify the expression of the different FLAG-TRIM5α proteins using an anti-FLAG antibody (upper panel) and an anti-GAPDH antibody as a control (bottom panel). **D.** MDTF (upper panel) and CRFK (bottom panel) cells stably expressing an empty vector, and Myc-tagged wild-type human TRIM5α or K10R, SIM1mut, SIM2mut and SIM3mut mutants were generated. The presence of the TRIM5α-Myc proteins was detected by Western blot using a Myc-antibody; the presence of actin was used as a control. **E.** The MDTF empty vector control cell line and the indicated TRIM5α-Myc overexpressing cell lines were mock treated or infected with increasing amounts of N-MLV luc. Forty-eight hours after infection, luciferase activity was measured. One representative experiment of four independent experiments is shown. **F.** The CRFK empty vector control and the various TRIM5α-Myc overexpressing cell lines were mock treated or infected with increase dose of N-MLV luc. Forty-eight hours after infection luciferase activity was measured. One representative experiment of three independent experiments is shown. Error bars indicate standard deviation among triplicates in the same experiment.

There is a complex relationship between the SUMO and ubiquitin pathways, and it is possible that the ubiquitin ligase activity of the RING domain of TRIM5α is required for N-MLV restriction upon SUMO-1 overexpression. To test this, we generated a mutant version of TRIM5α in which cystein 15 and 18 in the first zing finger were mutated to alanine (C15A/C18A), in the context of the FLAG-TRIM5α vector. The SUMO-1 block to N-MLV was restored when we reintroduced the C15A/C18A TRIM5α in the HA-SUMO-1/shRNA4 cell line (Figure 5C). This result indicated that the RING domain E3 ubiquitin ligase activity of TRIM5α is not required for the block of N-MLV infection upon SUMO-1 overexpression.

### SUMO-interacting motifs present in human TRIM5α are important for restriction of N-MLV

As our data suggested an unanticipated relationship between SUMO-1 and TRIM5α, we asked if TRIM5α contains any amino acid motifs indicative of an interaction with the SUMO conjugation pathway. Analysis of the TRIM5α protein sequence revealed an N-terminal consensus site for SUMO conjugation and three potential SUMO-interacting motifs (SIM) in the B30.2 domain (Figure 5A), motifs often identified in proteins involved in non-covalent SUMO binding. The first potential SIM (SIM1) consists of an ILGV hydrophobic core that is followed by a cysteine residue. The second potential SIM (SIM2) consists of a VIGL hydrophobic core that is juxtaposed with two acidic amino acids (EE). The third potential SIM (SIM3) consists of an IVPL hydrophobic core that is followed by a Ser residue. To determine the importance of these sites to the antiviral activity of TRIM5α, we independently mutated the consensus SUMO conjugation site (K10R), the first SIM (SIM1mut), the second SIM (SIM2mut), and the third SIM (SIM3mut) in the context of the FLAG-TRIM5α vector (Figure 5B). We transiently transfected the HA-SUMO-1/shRNA4 cell line with constructs encoding these human TRIM5α variants and, twenty-four hours after transfection, infected the cells with N-MLV luc. Luciferase activity was measured forty-eight hours post-infection, and TRIM5α protein expression was confirmed by Western blotting (Figure 5C, bottom). Both wild-type TRIM5α and K10R mutant proteins were able to restore the block of N-MLV infection in the HA-SUMO-1/shRNA4 cell line (Figure 5C). This suggests that SUMO conjugation of TRIM5α at K10R is not required for restriction of N-MLV. Although SUMOylation of TRIM5α may occur, in spite of many efforts, we have not been able to demonstrate biochemically that human TRIM5α undergoes SUMO conjugation (data not shown). In contrast, the mutations in the first and second SIM sequences of TRIM5α (SIM1mut, SIM2mut) relieves the restriction activity (Figure 5C). The SIM3mut protein restricted N-MLV infection to levels similar to wild type and K10R TRIM5α. These results suggest that SIM1 and SIM2 motifs present in the B30.2 domain of TRIM5α are important for restriction activity of TRIM5α in the context of the 293T HA-SUMO-1 cell line.

We wondered if SUMO-1 overexpression impacted on TRIM5α expression levels. To answer this we transiently express FLAG-TRIM5α wild type or the mutant versions in the empty vector control cell line or the cells that express HA-SUMO1, and compared by Western blot the levels of TRIM5α expressed in the 2 cell lines. When SUMO-1 is overexpressed we found that there was only 1.2-fold more wild-type and SIM3mut TRIM5α than in the empty vector control cells, and that no change was observed for K10R, SIM1mut or SIM2mut TRIM5α (Figure S1A and B). Therefore, SUMO-1 overexpression has no impact on TRIM5α expression levels.

It has been documented that exogenous expression of human TRIM5α in permissive cells, such as Crandall feline kidney (CRFK) fibroblast or *Mus dunni* (MDTF) cells, imparts a block against N-MLV as potent as the one observed in human cells [12], [13], [58]. To determine the ability of TRIM5α mutants to inhibit infection of N-MLV in cells that do not overexpress SUMO-1 and are permissive for N-MLV infection, we generated MDTF and CRFK cells stably expressing either wild-type or mutant Myc-tagged versions of TRIM5α. The expression levels of the mutants were somewhat variable (Figure 5D), though TRIM5α protein levels have not been found to correlate with the strength of restriction in cells where TRIM5α is overexpressed [25], [58]. To measure the retroviral restriction activities of the TRIM5α wild-type, K10R, and SIM mutants, populations of MDTF and CRFK cells expressing these various TRIM5α proteins were infected with increasing doses of N-MLV luc, and the cultures were analyzed for luciferase activity. In MDTF cells, wild-type and K10R TRIM5α restricted N-MLV. Importantly, the SIM1mut and SIM2mut proteins were completely unable to mediate restriction of N-MLV (Figure 5E), and the SIM3mut showed modestly reduced restriction as compared to the wild-type TRIM5α. As in the wild type TRIM5α overexpressing cells, both K10R and SIM3mut, restricted N-MLV infection before reverse transcription (Figure S2). Similar phenotypes of restriction for the different TRIM5α mutant proteins were observed in CRFK cells (Figure 5F). Thus, SIM1 and SIM2 of human TRIM5α are crucial for N-MLV restriction in general, not only in the context of SUMO-1 overexpression.

**Figure 6:**
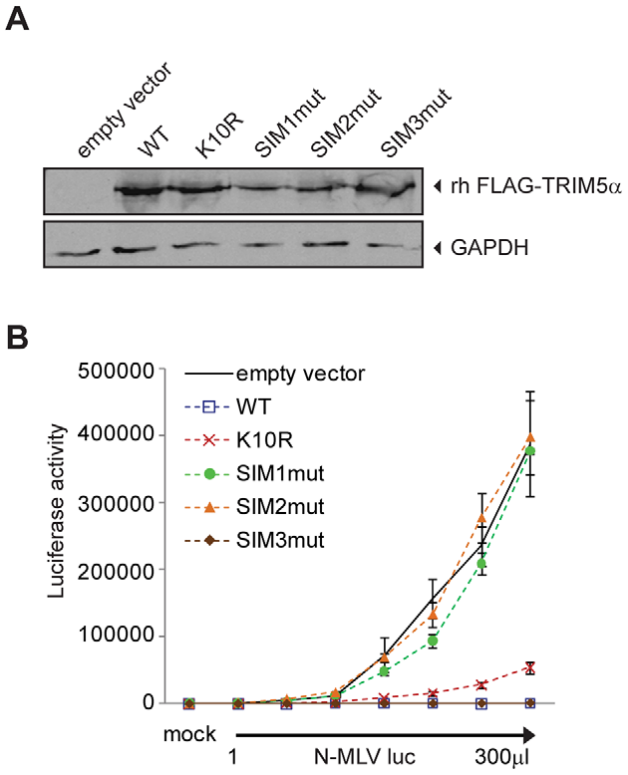
SUMO interacting motifs present in TRIM5α orthologs are important for restriction activity. **A**. CRFK cells stably expressing an empty vector, and FLAG-tagged wild-type rhesus TRIM5α or K10R, SIM1mut, SIM2mut and SIM3mut mutants were generated. The presence of the FLAG-TRIM5α proteins was detected by Western blot using a FLAG-antibody; the presence of GAPDH was used as a control. **B**. The CRFK empty vector control cell line and the indicated FLAG-TRIM5α overexpressing cell lines were mock treated or infected with increasing amounts of N-MLV luc. Forty-eight hours after infection, luciferase activity was measured. One representative experiment of three independent experiments is shown. Error bars indicate standard deviation among triplicates in the same experiment.

The SIMs present in human TRIM5α are conserved in several primates orthologs (Figure S3). To determine if the SIM1 and SIM2 are also required for restriction of N-MLV by TRIM5α of other species, we generated the same mutations used above in the rhesus monkey (*Macaca mulatta*) TRIM5α, which has been reported to restrict N-MLV infection [15]. We generated CRFK cell lines stably expressing either wild-type or mutant FLAG-tagged versions of rhesus TRIM5α, and the presence of the different rhesus TRIM5α proteins was detected by Western blot (Figure 6A). To measure the retroviral restriction activities of the rhesus TRIM5α wild-type, K10R, and SIM mutants, populations of CRFK cells expressing these various proteins were infected with increasing doses of N-MLV luc, and the cultures were analyzed for luciferase activity. Wild-type and SIM3mut TRIM5α restricted N-MLV. Mutant K10R showed modestly reduced restriction as compared to the wild-type TRIM5α. Most importantly, SIM1mut and SIM2mut rhesus TRIM5α were completely unable to mediate restriction of N-MLV (Figure 6B). These results suggest that SIM1 and SIM2 are important for restriction activity of different TRIM5α orthologs.

When expressed from transgenes, TRIM5α has been reported to form large cytoplasmic bodies [11], [59] or to exhibit a diffuse reticular pattern in the cytoplasm [32]. Several groups have demonstrated full retroviral restriction activity of TRIM5α in the absence of detectable cytoplasmic bodies [24], [32], [59], while others have shown the bodies to be highly dynamic structures that are key intermediates in the restriction process [60], [61]. We wondered if the mutations we introduced in TRIM5α could be altering its subcellular localization and if this possible change was the cause of the defective restriction observed. The CRFK cell lines expressing the TRIM5α wild-type or the different mutants were examined by immunofluorescence using a confocal microscope. In all cases, a punctate cytoplasmic staining pattern was observed with no difference between wild-type and the different mutant proteins (Figure S4). Therefore, the mutations introduced on TRIM5α are not altering its subcellular localization.

### CA mutations altering the SUMO conjugation sites reduce TRIM5α restriction

**Figure 7:**
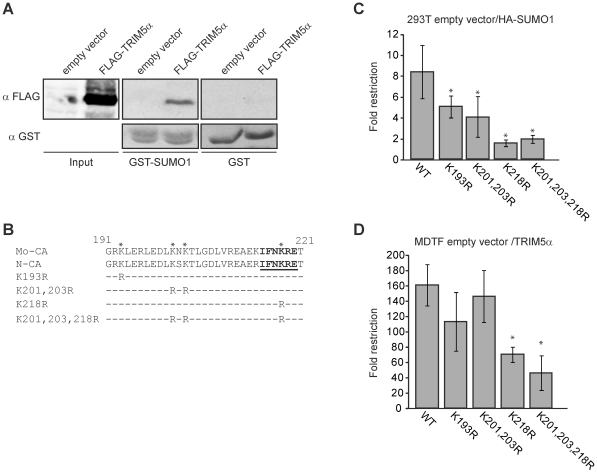
CA mutations altering putative SUMO conjugation site reduce SUMO-1 and TRIM5α-mediated restriction. **A.** The ability of human TRIM5α to interact with SUMO-1 is shown by GST-pulldown. 293T cell were transiently transfected with an empty plasmid or a plasmid encoding a FLAG-tagged version of human TRIM5α, forty-eight hours after transfection total extract were prepared and assayed for interaction with GST or GST-SUMO1 produced in bacteria. Input corresponds to 5% of the amount used in the interaction assay. **B.** Amino acid sequence of the UBC9 binding region of CA protein. The lysine residues conserved between Mo-CA and N-CA are highlighted, and the consensus binding sequence is underlined. The position and identity of the substituted amino acids in N-CA are indicated for each mutant. **C.** The 293T empty vector and HA-SUMO-1 cell lines were infected with wild type N-MLV luc or N-CA mutant viruses. **D.** MDTF empty vector and wild-type human TRIM5α cell lines were infected with wild-type N-MLV luc or the N-CA mutant viruses. Forty-eight hours after infection, luciferase activity was measured, and the fold restriction between the two cell lines was calculated for different experiments, presented as average fold restriction. Error bars indicate standard deviation among 6 different experiments. * indicates p<0.01.

The above results show that SIM1 and SIM2 in TRIM5α are required for restriction activity in the 293T SUMO-1-overexpressing and MDTF and CRFK TRIM5α overexpressing cell lines. We wondered if TRIM5α SIMs are required for binding SUMO-modified CA or another SUMO-modified cellular factor. First we tested if TRIM5α is able to bind SUMO-1. We transiently overexpressed FLAG-TRIM5α wild-type in 293T cells, and cellular lysates were used for a GST-pull down assay with GST or GST-SUMO-1 fusion proteins produced and purified from bacteria. We found that although weakly, TRIM5α is in fact, able to interact with SUMO-1 (Figure 7A).

Our laboratory has previously described that Mo-MLV CA interacts with the SUMO E2 ligase UBC9, and undergoes SUMO-1 conjugation. We have identified the domain of CA that interacts with UBC9 and PIAS4/y, and those lysine residues present in this domain and nearby that were required for CA SUMO conjugation [54]. To test whether these features of CA were involved in SUMO-1 restriction, we generated a series of mutant versions of N-MLV in which CA lysine residues in and near the UBC9 interaction were mutated to arginine (Figure 7B), analogous to mutations previously tested in the context of Mo-MLV. Although all the N-MLV luc mutant viruses showed reduced infectivity, as was reported for the equivalent mutants of Mo-MLV [54], they retained sufficient activity to allow us to infect cells, score for luciferase activity and observe the difference between restrictive and non restrictive cells (Figure S5).

We infected both the empty vector control and HA-SUMO-1-overexpressing 293T cell lines with various mutant N-MLV luc viruses at a range of multiplicities and calculated the fold restriction as the ratio between the luciferase activities of these cultures (Figure 7C and S5A). As previously observed, wild type N-MLV was restricted an average of 8-fold by SUMO-1 overexpression. The K193R and K201,203R N-MLV mutants were still restricted, but the restriction was reduced to 5- and 4-fold respectively. Strikingly the K218R and K201,203,218R mutant viruses were not significantly restricted (1.5 and 1.8 fold restriction respectively) by SUMO-1 overexpression. This result indicates that CA mutations altering the UBC9 interaction site, and lysines required for SUMO conjugation reduced the SUMO-1 block to N-MLV infection. Therefore, SUMO conjugation of CA is required for the SUMO-1-enhanced TRIM5α restriction activity.

To determine the effect of CA mutations on TRIM5α restriction when SUMO-1 is not overexpressed, we infected the MDTF empty vector and TRIM5α-overexpressing cell lines with either wild-type or CA mutant versions of N-MLV luc and measured the luciferase activity in culture lysates. The fold restriction was calculated as the ratio between the luciferase activities observed upon infection of the empty vector and TRIM5α cell lines. MTDF cells expressing TRIM5α restricted wild-type N-MLV by an average of 166 fold over the control cells. The K193R and K201,203R mutants were also profoundly restricted and had no significant reduction of restriction (p>0.1) when compared to wild-type virus. In contrast, the restriction of the K218R and K201,203,218R mutants was significantly, though only modestly, reduced (p<0.01) when compared to wild-type virus (71 and 50 fold respectively) (Figure 7D). Thus, mutation of the major UBC9 interaction site (K218) and the lysines required for SUMO conjugation of CA (K218, K201 and K203) reduced TRIM5α antiviral activity. These results, together with the evidence that the TRIM5α SIM1 and SIM2 are required for antiviral activity against N-MLV suggests that at least a portion of the antiviral activity of TRIM5α is mediated through the binding of its SIMs to SUMO-conjugated CA.

## Discussion

In this study, we have identified the involvement of the SUMO conjugation machinery in the TRIM5α mediated restriction of N-MLV. Our findings indicate that overexpression of SUMO-1 in 293T and TE671 cells enhances an intrinsic block to N-MLV infection of human cell lines (Figure 1C and 2C). We show that this enhanced block is dependent on virus tropism (Figure 3A), occurs before reverse transcription (Figure 3C), and can be abrogated by pre-infection with restricted virus (Figure 3B). These characteristics are shared by TRIM5α restriction of N-MLV in human cells, and by RNAi-mediated knockdown of TRIM5α in the SUMO-1 overexpressing cells, we confirmed that the enhanced block of N-MLV infection is due to TRIM5α activity (Figure 4C and D).

TRIM5α orthologues of primate and non-primate species have restrictive activity against a variety of retroviruses [62]–[65]. The four domains (RING, B-box, coiled-coil and B30.2) of TRIM5α have been extensively studied in efforts to elucidate their functions in retroviral restriction [21], [25], [27], [32], [66]–[68]. These studies have revealed the importance of both the RING and B30.2 domains of rhesus TRIM5α in the inhibition of HIV-1 infection [11] and the requirement of the RING, B-box and B30.2 domains of human TRIM5α in the inhibition of N-tropic MLV [32]. TRIM5α has been found to bind to the retroviral capsid via its B30.2 domain [25], [26], [58]. Several groups have proposed that TRIM5α leads to an acceleration of the viral uncoating process, which results in deleterious disassembly of the capsid structure and exposure of the viral RNA to destructive cellular factors [26], [64], [69]. There is also evidence that suggests a role for ubiquitination and proteasome-mediated degradation of CA in TRIM5α-mediated restriction [70], [71]. There has been no report indicating a relationship between the SUMO conjugation pathway and TRIM5α restriction activity. We consider that TRIM5α SUMO-1 conjugation could somehow enhance TRIM5α restriction activity. However, we have not been able to demonstrate SUMO-1 modification of TRIM5α, and in addition our data indicates that mutation of a potential SUMO conjugation site does not affect TRIM5α antiviral activity either in the 293T HA-SUMO-1 cell line (figure 5C) or in the TRIM5α-expressing MDTF (figure 5E) or CRFK cells (figure 5F), indicating that this is not the likely mechanism of enhanced antiviral activity of TRIM5α upon SUMO-1 overexpression. We also considered the possibility that SUMO-1 overexpression impacts TRIM5α expression levels. Although we cannot eliminate this possibility for the endogenous protein, an overexpressed FLAG-tagged TRIM5α had very similar levels of expression in control and HA-SUMO-1 overexpressing cells (Figure S1A), which argues against an enhancement of TRIM5α protein levels as the mechanism of increased activity upon SUMO-1 overexpression.

The ubiquitin and SUMO pathways have been described as having an antagonistic relationship, but further studies have revealed a more complex interplay between these pathways. There are multiple reports that SUMO conjugation can act as a signal to recruit E3 ubiquitin ligases, leading to proteasome-mediated degradation of the modified protein. To date, the so-called SUMO-targeted Ubiquitin ligase (STUbL) family has been described in yeast, *Dictyoselium*, *Drosophila*, and mammals. Notably, all known STUbL proteins have a RING finger domain and an active SIM, which mediates the noncovalent binding to SUMO [72], . Analysis of the TRIM5α protein sequence revealed three potential SIMs in the B30.2 domain. We found that mutation of the first two hydrophobic residues of SIM1 and SIM2 to lysine had dramatic effects on TRIM5α restriction of N-MLV. SIM1mut and SIM2mut versions of TRIM5α were not able to fully restore TRIM5α antiviral activity in the HA-SUMO-1/shRNA4 overexpressing cell line (Figure 5C) and had absolutely no restriction activity in MDTF and CRFK cells (Figure 5E and 5F respectively). The same was observed in the rhesus TRIM5α overexpressing cells (Figure 6B). Mutation of SIM3 had a more moderate effect on TRIM5α restriction; cells expressing SIM3mut were still able to restrict N-MLV, but not as effectively as the wild-type protein. These results are consistent with a model in which TRIM5α activity is partially mediated by SIMs binding to SUMO-modified CA.

If TRIM5α is a novel STUbL, it would explain our observations of the SUMO-1 antiviral effects in 293T cells and the current model of a proteasome-dependent TRIM5α-mediated restriction. SUMO-1 can be conjugated to Mo-CA in vivo [54], and the amino acid sequence of the SUMO conjugation site in N-CA is identical to that of Mo-CA. We can speculate that SUMO conjugation to N-CA facilitates or stabilize TRIM5α binding via the SIMs. With multiple SIMs, TRIM5α could either bind multiple SUMO-1 conjugated viral CA proteins present in the incoming virus, or one CA molecule with multiple SUMO modifications. Once TRIM5α binds to the SUMO-CA, the RING domain of TRIM5α could activate the proteasome-mediated degradation of CA or other viral proteins associated with it, leaving the viral RNA unprotected and vulnerable to cellular factors. It is also possible that the mere binding of TRIM5α to the SUMO-modified CA is sufficient to interfere with the viral life cycle, which could explain why in the SUMO-1 overexpresing cell line, a RING domain mutant is able to complement as efficiently as the wild-type protein the restriction observed upon SUMO-1 overexpression.

TRIM5α belong to a large family of TRIM proteins that were originally observed to oligomerize into high-order structures localizing to specific compartments in the cytoplasm and nucleus [75]. TRIM19, also known as PML, forms the so-called PML nuclear bodies, which are important host antiviral defense against DNA virus (for review see [76]). PML was shown to be SUMOylated on 3 lysine residues and also contain a SIM. Accordingly intramolecular interactions between the PML SUMO and SIM were proposed to underlie PML nuclear bodies formation and recruitment of partners [77], [78]. Although an appealing model, specific PML isoforms that do not contain the SIM are able to form normal bodies [79] and still have antiviral activity [80]. When expressed from transgenes, TRIM5α has been reported to form large cytoplasmic bodies [11], [59]. We observe that TRIM5α mutants that lack the SIM are still able to form cytosolic bodies, which is similar to the case of the PML protein that do not contain a SIM, but in our case this TRIM5α mutant proteins lack the antiviral activity. TRIM5α bodies have been described as highly dynamic structures that interact with cytoplasmic viral complexes [60], [81], and it could be possible that the mutations we have introduced are affecting the dynamics of these structures, making them incapable to get to the viral complexes. Also, if TRIM5α cytosolic bodies are highly dynamic structures, the presence of the SIMs may stabilize the binding to the incoming viral cores, which are primarily recognized through the N tropism determinants (arginine 110) on CA. It could be interesting in further studies analyze the localization of TRIM5α SIM mutants and viral complexes upon infection.

Our data showing that viruses with CA mutations in the SUMO conjugation site and nearby lysines are not fully restricted support the theory that TRIM5α SIMs bind SUMO-CA. More importantly, mutant viruses containing the K218R mutation, which removes the lysine in the major UBC9 interaction site, are poorly restricted relative to wild-type virus in some settings (Figure 7C and 7D). While these mutants are still restricted in the MDTF-TRIM5α cells (Figure 7D), this could be due to other lysines acting as acceptor sites for SUMO conjugation, and to the high levels of TRIM5α expression. Portions of TRIM5α in the B30.2 domain have been described as important for binding CA [26], [58], and TRIM5α may be able to bind CA molecules with reduced efficiency in the absence of SUMOylation. The SUMO-conjugation sites of CA are located in the C-terminal domain of the protein, and upon maturation the C-terminal domain of CA migrates to fill the interstitial spaces between hexamers in the N-terminal domain layer, in the current model of MLV CA structure (Burns and Goff unpublished data and [82]). This rearrangement of CA could allow the SUMOylated lysines of CA to be exposed in the surface of the incoming virus. It is possible that the TRIM5α protein can recognize the arginine residue 110 of N-CA and the SUMOylated lysines at the same time or that different TRIM5α molecules can independently recognize each feature. Unfortunately the exact location of the SUMO-conjugation sites of CA in the mature lattice is still not available.

Earlier work has suggested that the SUMO modification of CA is important for successful infection: MLV mutant viruses with alterations in the UBC9 interaction site or mutations that abolish SUMO conjugation are blocked in an early step of infection [54]. If SUMO conjugation of CA is an important step during viral infection, is likely that innate immunity might take advantage of this process to restrict viral infection. The presence of SIMs in TRIM5α could be an adaptative advantage of this protein for its antiviral activity. SIM1 and SIM2, which do have a role in human and rhesus TRIM5α restriction of N-MLV, are located outside of the variable regions, while SIM3 which does not have a role on restriction activity is inside of the variable region 3. The location of SIM1 and SIM2 suggest that they are important for TRIM5α function and in fact several TRIM5α orthologs contain the SIMs present in the human protein (Figure S3).

In conclusion, our data show that the SIMs of TRIM5α are important for restriction of N-MLV, and that their mutation abolishes antiviral activity. The presence of an intact UBC9 interaction site and lysines that potentially can be SUMO modified in N-CA are required for full restriction by human TRIM5α. We propose that TRIM5α SIMs increase affinity of recognition of SUMO-modified CA. Further analyses are required to determine if TRIM5α is indeed restricting virus as a STUbL.

## Materials and Methods

### Cell lines

Human embryonic fibroblast (293T), *Mus dunni* tail fibroblast (MDTF), human medulloblastoma cell line TE671 and Crandall feline kidney (CRFK) fibroblast were maintained in Dulbeccos's modified Eagle medium supplemented with 10% fetal bovine serum, 100 UI/ml penicillin and 100 mg/ml streptomycin. All cells were cultured at 37°C in 5% CO_2_.

### Plasmids, subcloning and mutagenesis

pCIG3-N and pCIG3-B express *gag* and *pol* from N-MLV and B-MLV respectively [83]. pCMVI expresses *gag* and *pol* from NB-MLV. p8.91 encodes *gag* and *pol* of HIV-1. pMD.G expresses the vesicular stomatitis virus envelope glycoprotein [21]. pFBLuc (Stratagene) is a reporter plasmid containing the firefly luciferase coding sequence flanked by MLV-based LTRs. pCNCG is a CMV-driven reporter plasmid containing the green fluorescent protein (GFP) coding sequence flanked by MLV-based LTRs. pQCXIH Retroviral vector (Clontech) is a bicistronic expression vector that expresses an inserted gene along with the hygromicin selection marker. pQCXIP Retroviral vector (Clontech) is a bicistronic expression vector that expresses an inserted gene along with the puromycin selection marker. pcDNA3xFLAG is a CMV-driven expression vector that allows expression of N-terminal FLAG epitope proteins [84]. SUMO-1 was subcloned from pSG5 His-SUMO-1 (gift from Dr. Anne Dejean of the Institut Pasteur). SUMO-2 and SUMO-3 were subcloned from pCDNA4 HisMaxC-SUMO-2 or HisMaxC-SUMO-3 (gifts from Dr. Yoshiaki Azuma, The University of Kansas) into pQCXIH, such that the His tag was replaced with the HA epitope. Human and rhesus TRIM5α were subcloned from pMIP-TRIM5α (gift from Dr. Jeremy Luban) [85] into pcDNA3xFLAG. Human TRIM5α K10R and SIMs substitution mutants were generated by two-step overlapping PCR and cloned into pcDNA3xFLAG. Human TRIM5α wild type, K10R and SIMs mutants were subcloned from pcDNA3xFLAG-TRIM5α vectors into pQCXIP, such that the N-terminal FLAG epitope was replaced with a C-terminal Myc epitope. FLAG rhesus TRIM5α wild type, K10R and SIMs mutants were subcloned from pcDNA3xFLAG-TRIM5α vectors into pQCXIP. The pCIG3-N mutant versions CA R110E, CA K193R, CA K201,203R, CA K218R and CA K201,203,218R were generated by site-directed mutagenesis using Quick Change Lightning kit (Stratagene). Note: All primer sequences are available upon request.

### Generation of stable cell lines

Retroviruses for transduction were produced by transfection of 293T cells with 1 µg pMD.G, 1 µg pCMVI and 1.5 µg of either pQCXIH, pQCXIH-HA-SUMO-1, pQCXIP, pQCXIP-TRIM5α wild-type or mutant versions, using FUGENE (Roche). Viruses were harvested 48 h after transfection, filtered (0.45 µm) and used to infect 5×10^5^ cells in 100 mm dishes in the presence of 8 µg/ml polybrene. 293T, MDTF and TE671 cells infected with vectors delivering the hygro^r^ gene were selected in 200 µg/ml hygromycin. Cells infected with vectors containing the puro^r^ gene were selected either in 5 µg/ml puromycin (MDTF cells) or in 7.5 µg/ml puromycin (CRFK cells). Lentiviruses for transduction were produced by transfection of 293T cells with 1 µg pMD.G, 1 µg p8.91 and 1.5 µg of pGIPz (Open Biosystems) or pGIPzTRIM5α DNAs containing shRNAs #1 to #5 (Open Biosystems). Viruses were harvested 48 h after infection, filtered (0.45 µm) and used to infect 5×10^4^ 293T HA-SUMO-1 cells in 35 mm dishes in the presence of 8 µg/ml polybrene. Cells were selected in 200 µg/ml hygromycin and 1.5 µg/ml puromycin.

### Western blotting

Cells were lysed in 20 mM Tris-HCl (pH 8.0), 137 mM KCl, 10% glycerol, 1% NP-40 and Complete protease inhibitor (Roche) or Reporter lysis buffer (Promega). Samples were then boiled in 5× sodium dodecyl sulphate (SDS) loading buffer, and the proteins were resolved by SDS-polyacrylamide gel electrophoresis (PAGE). After transfer to nitrocellulose membranes, the blots were probed with mouse anti-β actin (Sigma), mouse anti-HA (Covance), mouse anti-FLAG (Sigma) or mouse anti-c-Myc (Santa Cruz Biotechnology).

### Single-cycle infectivity assay

B-, NB- and N-tropic luciferase reporter viruses were produced by transfection of 293T cells with 1 µg pCIG3-B or pCMVI or pCIG3-N (the wild type or mutant versions), 1 µg pMD.G and 1.5 µg pFBluc or pCNCG (per 100 mm plate) using FUGENE (Roche). Reporter virus stocks were harvested 48 h after transfection, filtered (0.45 µm) and stored at −80°C. 293T (3×10^4^ per well), MDTF (2.5×10^4^ per well), TE671 (2.5×10^4^ per well) and CRFK (3×10^4^ per well) cells were seeded in 24-well plates and infected with MLV luc reporter viruses. For reactions involving transient transfections, cells were transfected with 100 ng of pcDNA3xFLAG or pcDNA3xFLAG-TRIM5α wild type or mutant versions. Twenty-four hours post-transfection, cells were infected with N-MLV luc virus. Forty-eight hours post-infection cells were collected and assayed for firefly luciferase activity (Promega) in a luminometer.

### Analysis of viral DNA synthesized in vivo

Cells 293T (1×10^5^) plated in 35 mm dishes were infected with N-MLV luc for six hours. Twenty-four hours post-infection, cells were trypsinized, pelleted and total DNA was collected using DNeasy Qiagen kit (Qiagen). Quantitative PCR (qPCR) analysis was performed using primers to amplify the minus-strand strong stop (MSS), 2-LTR circles (LTR-LTR junction) and luciferase DNA as previously described [86].

### Analysis of TRIM5α knock down

Cells were harvested and total RNA was extracted using TRIZOL reagent (Invitrogen). 2 µg of total RNA per cell line was used for reverse transcription reactions to produce cDNA using random hexamers and SuperScript III kit (Invitrogen). 2 µl of each cDNA was used for qPCR analysis of TRIM5α and GAPDH transcript levels. Fold change was calculated using the relative standard curve method.

### GST-pull down assay

GST and the fusion protein GST-SUMO1 were produce in *Escherichia coli* BL-21 (DE3) as previously described [87]. 293T cells were transfected with 3 µg of pcDNA3xFLAG-humanTRIM5α or the empty vector. Forty-eight hours after transfection the cells were lysed on 20 mM Tris-HCl (pH 8.0), 137 mM KCl, 10% glycerol, 1% NP-40 and Complete protease inhibitor (Roche) 20 minutes at 4°C, the lysate was clarified by centrifugation at 13000×g for 10 minutes at 4°C. GST-pulldown assays were performed using 100 µl of cell lysate and 2 µg of purified GST or GST-SUMO1 and 20 µl of Glutathione Sepharose 4B (Amersham biosciences) in binding buffer (20 mM Tris-HCl (pH 8.0), 100 mM KCl, 10 mM EDTA, 0.5 mM DTT, Complete protease inhibitor) for 2 hours, the beads were washed 4 times with wash buffer (20 mM Tris-HCl (pH 8.0), 100 mM KCl, 0.2% NP-40 10 mM EDTA, 0.5 mM DTT, Complete protease inhibitor), the beads were resuspended with 40 µl of GLB2x, and the proteins were resolved by SDS-PAGE. After transfer to nitrocellulose membranes, the blots were probed with mouse anti-FLAG (Sigma) or mouse anti-GST (Covance).

### Accession numbers

SUMO-1: AACC5096.1

SUMO-2: P61956.2

SUMO-3: NP_008867.2

MoMLV: AF033811

N-tropic MLV gag-pol region: K01203.1

B-tropic MLV gag-pol region: K01204.1

TRIM5α: *H.sapiens* ABB90543, *M. mulatta* NP_0010228082

## Supporting Information

Figure S1 SUMO-1 overexpression does not modified TRIM5α protein levels. **A.** The 293T empty vector or HA-SUMO-1 overexpressing cells were transiently transfected with 100 ng of an empty plasmid or plasmids encoding FLAG-tagged human TRIM5α wild-type or the K10R, SIM1mut, SIM2mut and SIM3mut versions. Forty-eight hours after transfection the cells were lysed and the presence of the different TRIM5α proteins was assayed by Western blot using an anti-FLAG antibody. The presence of GAPDH was used as loading control. **B.** The levels of the FLAG-TRIM5α proteins in the HA-SUMO-1 cell line were quantified and expressed as relative values to the levels of FLAG-TRIM5α in the control cell line; in both cases the protein level was normalized to GAPDH. Error bars correspond to standard deviation from 4 independent experiments.
(TIF)

Figure S2 K10R and SIM3 mutant block N-MLV infection before reverse transcription. The MDTF empty vector control or human TRIM5α wild-type, K10R or SIM3mut overexpressing cell lines were infected with VSV-G-pseudotyped N-MLV luc. Low molecular weight DNA was isolated twenty hours after infection, and the amount of viral DNA synthesized in the infected cells was measured by quantitative PCR. Primers specific for the minus-strand strong stop (MSS) DNA (black bars), Luciferase gene (grey bars) or LTR-LTR junction (white bars) were used. The values were normalized to mitochondrial DNA and expressed as fold over empty vector. Error bars indicate standard deviation from 3 different experiments.
(TIF)

Figure S3 SUMO interacting motifs of TRIM5α are conserved in primate orthologs. An alignment of the amino acid sequence of TRIM5α B30.2 domain from several primates orthologs is shown. The three variable regions (V1–V3) are depicted with horizontal bars above the alignment. The SIMs are indicated in red letters. Dashes represent gaps. The sequences were retrieved from protein data bank and aligned using AlignX (Invitrogen). *H.sapiens* (ABB90543), *P. troglodytes* (AAV91977), *P. pygmaeus* (AAV91984), *G. gorilla* (AAV91981), *S. syndactylus* (AAV91980), *M. mulatta* (NP_0010228082), *C. guereza* (AAV91978), *P. nemaeus* (AAV91979), *E. patas* (V91985), *M. fascicularis* (BAD93339), *C. tantalus* (AAT10388), *C. aethiops* (AAV91975), *P. Anubis* (AAV91976), *A. geoffroyi* (AAV91987), *L. logotricha* (Q5D7H7), *P. pithecia* (AAV91986), *C. donacophilus* (AAV919990), *S. sciureus* (AAV919888), *S. labiatus* (AAV91989).
(TIF)

Figure S4 Mutations in TRIM5α do not change its subcellular localization. The subcellular localization of rhesus TRIM5α stably expressed in CRFK cells was assayed. Empty vector control or TRIM5α overexpressing cells were grown on coverslips for twenty-four hours and fixed in 3.7% formaldehyde in PBS. After permeabilization with 1% triton X-100 the cells were incubated with anti-FLAG M2 monoclonal antibody (Sigma) at a dilution of 1∶500 and a secondary anti-mouse conjugated with Alexa Fluor-488 (Molecular probes) at a dilution of 1∶500. Vectashield mounting medium with DAPI (Vector) was used. The cells were visualized with a UPLSAPO 60× 1.35-numerical aperture Olympus oil immersion objective using a Olympus BX61 microscope fitted with a FV1000 FLUO laser scanning confocal system. Image analysis was performed using MethaMorph software.
(TIF)

Figure S5 CA mutations altering putative SUMO conjugation site reduce viral infectivity. **A.** The 293T empty vector and HA-SUMO-1 cell lines were infected with wild type N-MLV luc or N-CA mutant viruses. **B.** MDTF empty vector and wild-type human TRIM5α cell lines were infected with wild-type N-MLV luc or the N-CA mutant viruses. Forty-eight hours after infection, luciferase activity was measured. One representative of 6 different experiments is shown. Error bars indicate standard deviation of triplicates in the same experiment.
(TIF)

## References

[1] Wolf D, Goff SP (2008). Host restriction factors blocking retroviral
replication.. Annu Rev Genet.

[2] Lilly F (1967). Susceptibility to two strains of Friend leukemia virus in
mice.. Science.

[3] Best S, Le Tissier P, Towers G, Stoye JP (1996). Positional cloning of the mouse retrovirus restriction gene
Fv1.. Nature.

[4] Hartley JW, Rowe WP, Huebner RJ (1970). Host-range restrictions of murine leukemia viruses in mouse
embryo cell cultures.. J Virol.

[5] Hopkins N, Schindler J, Hynes R (1977). Six-NB-tropic murine leukemia viruses derived from a B-tropic
virus of BALB/c have altered p30.. J Virol.

[6] DesGroseillers L, Jolicoeur P (1983). Physical mapping of the Fv-1 tropism host range determinant of
BALB/c murine leukemia viruses.. J Virol.

[7] Kozak CA, Chakraborti A (1996). Single amino acid changes in the murine leukemia virus capsid
protein gene define the target of Fv1 resistance.. Virology.

[8] Rowe WP (1972). Studies of genetic transmission of murine leukemia virus by AKR
mice. I. Crosses with Fv-1 n strains of mice.. J Exp Med.

[9] Jolicoeur P, Baltimore D (1976). Effect of Fv-1 gene product on proviral DNA formation and
integration in cells infected with murine leukemia viruses.. Proc Natl Acad Sci U S A.

[10] Boone LR, Innes CL, Heitman CK (1990). Abrogation of Fv-1 restriction by genome-deficient virions
produced by a retrovirus packaging cell line.. J Virol.

[11] Stremlau M, Owens CM, Perron MJ, Kiessling M, Autissier P (2004). The cytoplasmic body component TRIM5alpha restricts HIV-1
infection in Old World monkeys.. Nature.

[12] Hatziioannou T, Perez-Caballero D, Yang A, Cowan S, Bieniasz PD (2004). Retrovirus resistance factors Ref1 and Lv1 are species-specific
variants of TRIM5alpha.. Proc Natl Acad Sci U S A.

[13] Keckesova Z, Ylinen LM, Towers GJ (2004). The human and African green monkey TRIM5alpha genes encode Ref1
and Lv1 retroviral restriction factor activities.. Proc Natl Acad Sci U S A.

[14] Perron MJ, Stremlau M, Song B, Ulm W, Mulligan RC (2004). TRIM5alpha mediates the postentry block to N-tropic murine
leukemia viruses in human cells.. Proc Natl Acad Sci U S A.

[15] Yap MW, Nisole S, Lynch C, Stoye JP (2004). Trim5alpha protein restricts both HIV-1 and murine leukemia
virus.. Proc Natl Acad Sci U S A.

[16] Besnier C, Takeuchi Y, Towers G (2002). Restriction of lentivirus in monkeys.. Proc Natl Acad Sci U S A.

[17] Besnier C, Ylinen L, Strange B, Lister A, Takeuchi Y (2003). Characterization of murine leukemia virus restriction in
mammals.. J Virol.

[18] Cowan S, Hatziioannou T, Cunningham T, Muesing MA, Gottlinger HG (2002). Cellular inhibitors with Fv1-like activity restrict human and
simian immunodeficiency virus tropism.. Proc Natl Acad Sci U S A.

[19] Himathongkham S, Luciw PA (1996). Restriction of HIV-1 (subtype B) replication at the entry step in
rhesus macaque cells.. Virology.

[20] Towers G, Bock M, Martin S, Takeuchi Y, Stoye JP (2000). A conserved mechanism of retrovirus restriction in
mammals.. Proc Natl Acad Sci U S A.

[21] Yap MW, Nisole S, Stoye JP (2005). A single amino acid change in the SPRY domain of human Trim5alpha
leads to HIV-1 restriction.. Curr Biol.

[22] Nisole S, Stoye JP, Saib A (2005). TRIM family proteins: retroviral restriction and antiviral
defence.. Nat Rev Microbiol.

[23] Nakayama EE, Miyoshi H, Nagai Y, Shioda T (2005). A specific region of 37 amino acid residues in the SPRY (B30.2)
domain of African green monkey TRIM5alpha determines species-specific
restriction of simian immunodeficiency virus SIVmac
infection.. J Virol.

[24] Perez-Caballero D, Hatziioannou T, Zhang F, Cowan S, Bieniasz PD (2005). Restriction of human immunodeficiency virus type 1 by TRIM-CypA
occurs with rapid kinetics and independently of cytoplasmic bodies,
ubiquitin, and proteasome activity.. J Virol.

[25] Sebastian S, Luban J (2005). TRIM5alpha selectively binds a restriction-sensitive retroviral
capsid.. Retrovirology.

[26] Stremlau M, Perron M, Lee M, Li Y, Song B (2006). Specific recognition and accelerated uncoating of retroviral
capsids by the TRIM5alpha restriction factor.. Proc Natl Acad Sci U S A.

[27] Stremlau M, Perron M, Welikala S, Sodroski J (2005). Species-specific variation in the B30.2(SPRY) domain of
TRIM5alpha determines the potency of human immunodeficiency virus
restriction.. J Virol.

[28] Diaz-Griffero F, Li X, Javanbakht H, Song B, Welikala S (2006). Rapid turnover and polyubiquitylation of the retroviral
restriction factor TRIM5.. Virology.

[29] Massiah MA, Matts JA, Short KM, Simmons BN, Singireddy S (2007). Solution structure of the MID1 B-box2 CHC(D/C)C(2)H(2)
zinc-binding domain: insights into an evolutionarily conserved RING
fold.. J Mol Biol.

[30] Berthoux L, Sebastian S, Sayah DM, Luban J (2005). Disruption of human TRIM5alpha antiviral activity by nonhuman
primate orthologues.. J Virol.

[31] Mische CC, Javanbakht H, Song B, Diaz-Griffero F, Stremlau M (2005). Retroviral restriction factor TRIM5alpha is a
trimer.. J Virol.

[32] Perez-Caballero D, Hatziioannou T, Yang A, Cowan S, Bieniasz PD (2005). Human tripartite motif 5alpha domains responsible for retrovirus
restriction activity and specificity.. J Virol.

[33] Hay RT (2005). SUMO: a history of modification.. Mol Cell.

[34] Geiss-Friedlander R, Melchior F (2007). Concepts in sumoylation: a decade on.. Nat Rev Mol Cell Biol.

[35] Desterro JM, Thomson J, Hay RT (1997). Ubch9 conjugates SUMO but not ubiquitin.. FEBS Lett.

[36] Schwarz SE, Matuschewski K, Liakopoulos D, Scheffner M, Jentsch S (1998). The ubiquitin-like proteins SMT3 and SUMO-1 are conjugated by the
UBC9 E2 enzyme.. Proc Natl Acad Sci U S A.

[37] Hochstrasser M (2001). SP-RING for SUMO: new functions bloom for a ubiquitin-like
protein.. Cell.

[38] Jackson PK (2001). A new RING for SUMO: wrestling transcriptional responses into
nuclear bodies with PIAS family E3 SUMO ligases.. Genes Dev.

[39] Verger A, Perdomo J, Crossley M (2003). Modification with SUMO. A role in transcriptional
regulation.. EMBO Rep.

[40] Saitoh H, Hinchey J (2000). Functional heterogeneity of small ubiquitin-related protein
modifiers SUMO-1 versus SUMO-2/3.. J Biol Chem.

[41] Rosas-Acosta G, Russell WK, Deyrieux A, Russell DH, Wilson VG (2005). A universal strategy for proteomic studies of SUMO and other
ubiquitin-like modifiers.. Mol Cell Proteomics.

[42] Vertegaal AC, Andersen JS, Ogg SC, Hay RT, Mann M (2006). Distinct and overlapping sets of SUMO-1 and SUMO-2 target
proteins revealed by quantitative proteomics.. Mol Cell Proteomics.

[43] Minty A, Dumont X, Kaghad M, Caput D (2000). Covalent modification of p73alpha by SUMO-1. Two-hybrid screening
with p73 identifies novel SUMO-1-interacting proteins and a SUMO-1
interaction motif.. J Biol Chem.

[44] Sampson DA, Wang M, Matunis MJ (2001). The small ubiquitin-like modifier-1 (SUMO-1) consensus sequence
mediates Ubc9 binding and is essential for SUMO-1
modification.. J Biol Chem.

[45] Hannich JT, Lewis A, Kroetz MB, Li SJ, Heide H (2005). Defining the SUMO-modified proteome by multiple approaches in
Saccharomyces cerevisiae.. J Biol Chem.

[46] Song J, Zhang Z, Hu W, Chen Y (2005). Small ubiquitin-like modifier (SUMO) recognition of a SUMO
binding motif: a reversal of the bound orientation.. J Biol Chem.

[47] Hecker CM, Rabiller M, Haglund K, Bayer P, Dikic I (2006). Specification of SUMO1- and SUMO2-interacting
motifs.. J Biol Chem.

[48] Boggio R, Chiocca S (2006). Viruses and sumoylation: recent highlights.. Curr Opin Microbiol.

[49] Boggio R, Colombo R, Hay RT, Draetta GF, Chiocca S (2004). A mechanism for inhibiting the SUMO pathway.. Mol Cell.

[50] Parkinson J, Everett RD (2000). Alphaherpesvirus proteins related to herpes simplex virus type 1
ICP0 affect cellular structures and proteins.. J Virol.

[51] Lamsoul I, Lodewick J, Lebrun S, Brasseur R, Burny A (2005). Exclusive ubiquitination and sumoylation on overlapping lysine
residues mediate NF-kappaB activation by the human T-cell leukemia virus tax
oncoprotein.. Mol Cell Biol.

[52] Gurer C, Berthoux L, Luban J (2005). Covalent modification of human immunodeficiency virus type 1 p6
by SUMO-1.. J Virol.

[53] Weldon RA, Sarkar P, Brown SM, Weldon SK (2003). Mason-Pfizer monkey virus Gag proteins interact with the human
sumo conjugating enzyme, hUbc9.. Virology.

[54] Yueh A, Leung J, Bhattacharyya S, Perrone LA, de los Santos K (2006). Interaction of moloney murine leukemia virus capsid with Ubc9 and
PIASy mediates SUMO-1 addition required early in infection.. J Virol.

[55] Aagaard L, Mikkelsen JG, Warming S, Duch M, Pedersen FS (2002). Fv1-like restriction of N-tropic replication-competent murine
leukaemia viruses in mCAT-1-expressing human cells.. J Gen Virol.

[56] Towers G, Collins M, Takeuchi Y (2002). Abrogation of Ref1 retrovirus restriction in human
cells.. J Virol.

[57] Shibata R, Sakai H, Kawamura M, Tokunaga K, Adachi A (1995). Early replication block of human immunodeficiency virus type 1 in
monkey cells.. J Gen Virol.

[58] Sebastian S, Grutter C, Strambio de Castillia C, Pertel T, Olivari S (2009). An invariant surface patch on the TRIM5alpha PRYSPRY domain is
required for retroviral restriction but dispensable for capsid
binding.. J Virol.

[59] Song B, Diaz-Griffero F, Park DH, Rogers T, Stremlau M (2005). TRIM5alpha association with cytoplasmic bodies is not required
for antiretroviral activity.. Virology.

[60] Campbell EM, Dodding MP, Yap MW, Wu X, Gallois-Montbrun S (2007). TRIM5 alpha cytoplasmic bodies are highly dynamic
structures.. Mol Biol Cell.

[61] Anderson JL, Campbell EM, Wu X, Vandegraaff N, Engelman A (2006). Proteasome inhibition reveals that a functional preintegration
complex intermediate can be generated during restriction by diverse TRIM5
proteins.. J Virol.

[62] Song B, Gold B, O'Huigin C, Javanbakht H, Li X (2005). The B30.2(SPRY) domain of the retroviral restriction factor
TRIM5alpha exhibits lineage-specific length and sequence variation in
primates.. J Virol.

[63] Song B, Javanbakht H, Perron M, Park DH, Stremlau M (2005). Retrovirus restriction by TRIM5alpha variants from Old World and
New World primates.. J Virol.

[64] Shi J, Aiken C (2006). Saturation of TRIM5 alpha-mediated restriction of HIV-1 infection
depends on the stability of the incoming viral capsid.. Virology.

[65] Ylinen LM, Keckesova Z, Webb BL, Gifford RJ, Smith TP (2006). Isolation of an active Lv1 gene from cattle indicates that
tripartite motif protein-mediated innate immunity to retroviral infection is
widespread among mammals.. J Virol.

[66] Javanbakht H, Diaz-Griffero F, Stremlau M, Si Z, Sodroski J (2005). The contribution of RING and B-box 2 domains to retroviral
restriction mediated by monkey TRIM5alpha.. J Biol Chem.

[67] Li X, Gold B, O'HUigin C, Diaz-Griffero F, Song B (2007). Unique features of TRIM5alpha among closely related human TRIM
family members.. Virology.

[68] Li X, Li Y, Stremlau M, Yuan W, Song B (2006). Functional replacement of the RING, B-box 2, and coiled-coil
domains of tripartite motif 5alpha (TRIM5alpha) by heterologous TRIM
domains.. J Virol.

[69] Perron MJ, Stremlau M, Lee M, Javanbakht H, Song B (2007). The human TRIM5alpha restriction factor mediates accelerated
uncoating of the N-tropic murine leukemia virus capsid.. J Virol.

[70] Diaz-Griffero F, Kar A, Perron M, Xiang SH, Javanbakht H (2007). Modulation of retroviral restriction and proteasome
inhibitor-resistant turnover by changes in the TRIM5alpha B-box 2
domain.. J Virol.

[71] Yamauchi K, Wada K, Tanji K, Tanaka M, Kamitani T (2008). Ubiquitination of E3 ubiquitin ligase TRIM5 alpha and its
potential role.. FEBS J.

[72] Prudden J, Pebernard S, Raffa G, Slavin DA, Perry JJ (2007). SUMO-targeted ubiquitin ligases in genome
stability.. EMBO J.

[73] Uzunova K, Gottsche K, Miteva M, Weisshaar SR, Glanemann C (2007). Ubiquitin-dependent proteolytic control of SUMO
conjugates.. J Biol Chem.

[74] Geoffroy MC, Hay RT (2009). An additional role for SUMO in ubiquitin-mediated
proteolysis.. Nat Rev Mol Cell Biol.

[75] Reymond A, Meroni G, Fantozzi A, Merla G, Cairo S (2001). The tripartite motif family identifies cell
compartments.. EMBO J.

[76] Tavalai N, Stamminger T (2008). New insights into the role of the subnuclear structure ND10 for
viral infection.. Biochim Biophys Acta.

[77] Matunis MJ, Zhang XD, Ellis NA (2006). SUMO: the glue that binds.. Dev Cell.

[78] Shen TH, Lin HK, Scaglioni PP, Yung TM, Pandolfi PP (2006). The mechanisms of PML-nuclear body formation.. Mol Cell.

[79] Weidtkamp-Peters S, Lenser T, Negorev D, Gerstner N, Hofmann TG (2008). Dynamics of component exchange at PML nuclear
bodies.. J Cell Sci.

[80] Reichelt M, Wang L, Sommer M, Perrino J, Nour AM (2011). Entrapment of Viral Capsids in Nuclear PML Cages Is an Intrinsic
Antiviral Host Defense against Varicella-Zoster Virus.. PLoS Pathog.

[81] Campbell EM, Perez O, Anderson JL, Hope TJ (2008). Visualization of a proteasome-independent intermediate during
restriction of HIV-1 by rhesus TRIM5alpha.. J Cell Biol.

[82] Mortuza GB, Dodding MP, Goldstone DC, Haire LF, Stoye JP (2008). Structure of B-MLV capsid amino-terminal domain reveals key
features of viral tropism, gag assembly and core formation.. J Mol Biol.

[83] Bock M, Bishop KN, Towers G, Stoye JP (2000). Use of a transient assay for studying the genetic determinants of
Fv1 restriction.. J Virol.

[84] Fu D, Collins K (2006). Human telomerase and Cajal body ribonucleoproteins share a unique
specificity of Sm protein association.. Genes Dev.

[85] Sebastian S, Sokolskaja E, Luban J (2006). Arsenic counteracts human immunodeficiency virus type 1
restriction by various TRIM5 orthologues in a cell type-dependent
manner.. J Virol.

[86] Haedicke J, de Los Santos K, Goff SP, Naghavi MH (2008). The Ezrin-radixin-moesin family member ezrin regulates stable
microtubule formation and retroviral infection.. J Virol.

[87] Arriagada G, Paredes R, van Wijnen AJ, Lian JB, van Zundert B (2010). 1alpha,25-dihydroxy vitamin D(3) induces nuclear matrix
association of the 1alpha,25-dihydroxy vitamin D(3) receptor in osteoblasts
independently of its ability to bind DNA.. J Cell Physiol.

