# Supplementary figures and images for: SUMO-Interacting Motifs of Human TRIM5α are Important for Antiviral Activity
Source: PLoS Pathog. 2011 Apr 7;7(4):e1002019. doi: 10.1371/journal.ppat.1002019 (PMC3072370; doi:10.1371/journal.ppat.1002019)

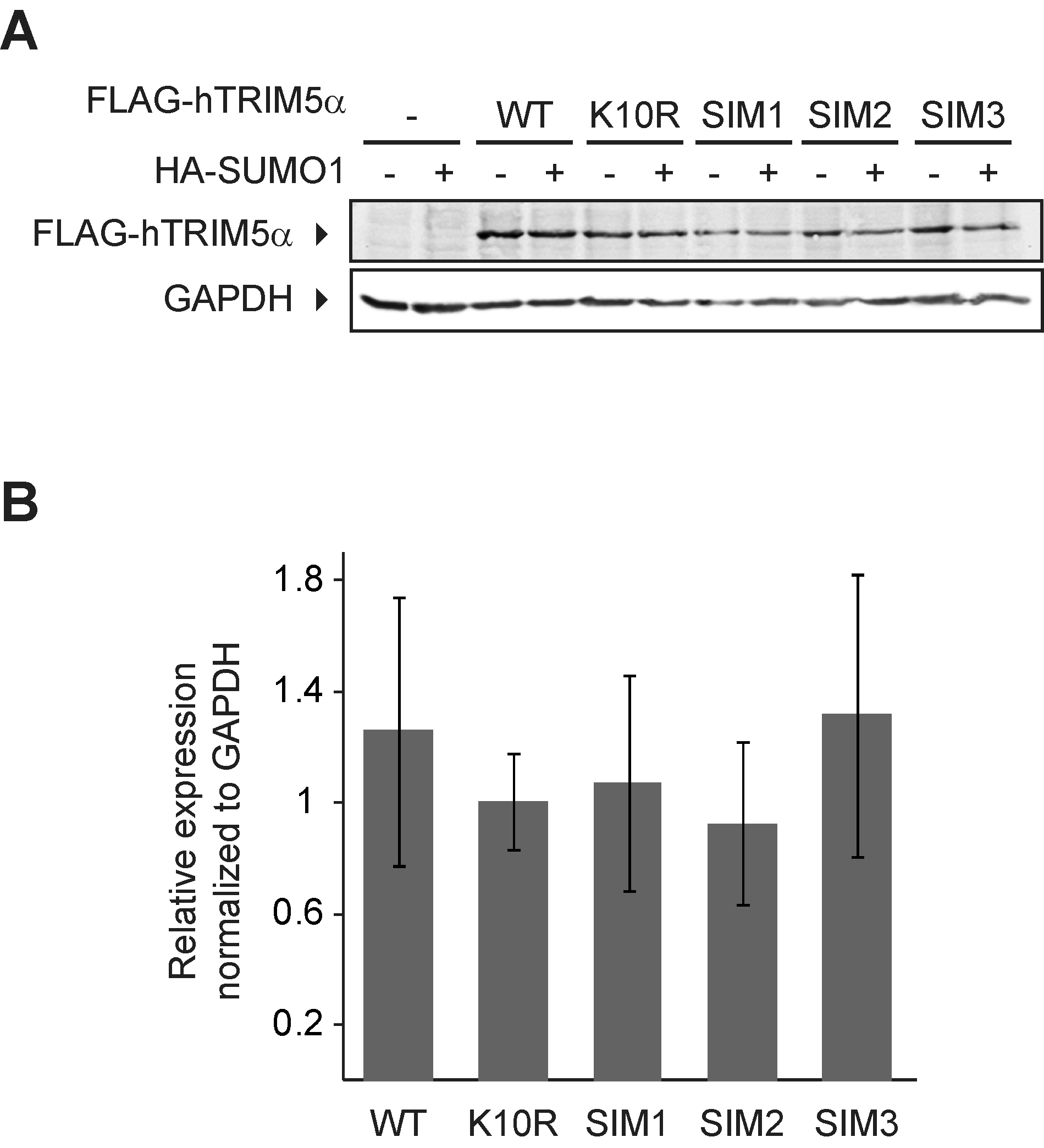

Supplement: Figure S1 — SUMO-1 overexpression does not modified TRIM5α protein levels. A. The 293T empty vector or HA-SUMO-1 overexpressing cells were transiently transfected with 100 ng of an empty plasmid or plasmids encoding FLAG-tagged human TRIM5α wild-type or the K10R, SIM1mut, SIM2mut and SIM3mut versions. Forty-eight hours after transfection the cells were lysed and the presence of the different TRIM5α proteins was assayed by Western blot using an anti-FLAG antibody. The presence of GAPDH was used as loading control. B. The levels of the FLAG-TRIM5α proteins in the HA-SUMO-1 cell line were quantified and expressed as relative values to the levels of FLAG-TRIM5α in the control cell line; in both cases the protein level was normalized to GAPDH. Error bars correspond to standard deviation from 4 independent experiments. (TIF) [file ppat.1002019.s001.tif]

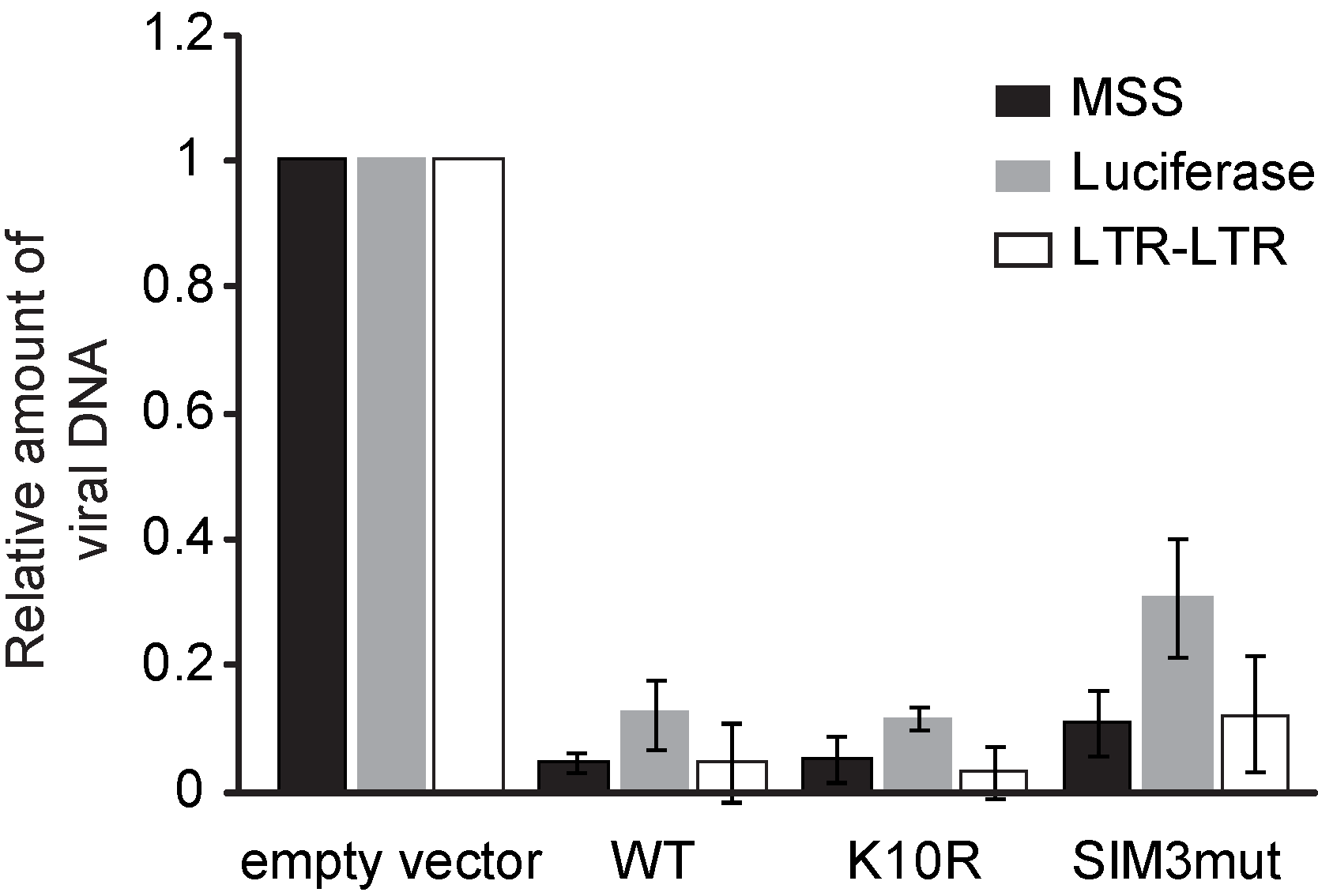

Supplement: Figure S2 — K10R and SIM3 mutant block N-MLV infection before reverse transcription. The MDTF empty vector control or human TRIM5α wild-type, K10R or SIM3mut overexpressing cell lines were infected with VSV-G-pseudotyped N-MLV luc. Low molecular weight DNA was isolated twenty hours after infection, and the amount of viral DNA synthesized in the infected cells was measured by quantitative PCR. Primers specific for the minus-strand strong stop (MSS) DNA (black bars), Luciferase gene (grey bars) or LTR-LTR junction (white bars) were used. The values were normalized to mitochondrial DNA and expressed as fold over empty vector. Error bars indicate standard deviation from 3 different experiments. (TIF) [file ppat.1002019.s002.tif]

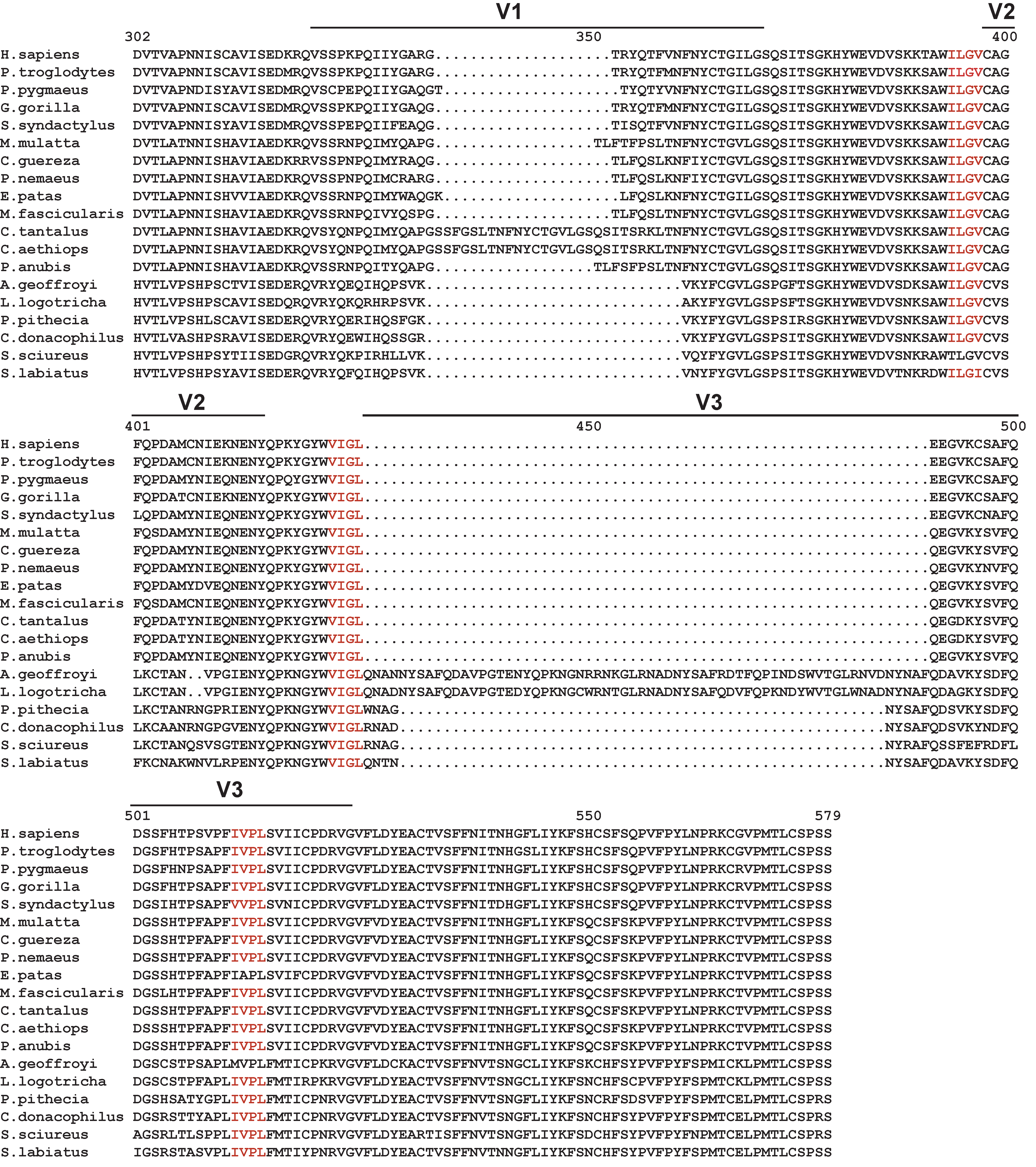

Supplement: Figure S3 — SUMO interacting motifs of TRIM5α are conserved in primate orthologs. An alignment of the amino acid sequence of TRIM5α B30.2 domain from several primates orthologs is shown. The three variable regions (V1–V3) are depicted with horizontal bars above the alignment. The SIMs are indicated in red letters. Dashes represent gaps. The sequences were retrieved from protein data bank and aligned using AlignX (Invitrogen). H.sapiens (ABB90543), P. troglodytes (AAV91977), P. pygmaeus (AAV91984), G. gorilla (AAV91981), S. syndactylus (AAV91980), M. mulatta (NP_0010228082), C. guereza (AAV91978), P. nemaeus (AAV91979), E. patas (V91985), M. fascicularis (BAD93339), C. tantalus (AAT10388), C. aethiops (AAV91975), P. Anubis (AAV91976), A. geoffroyi (AAV91987), L. logotricha (Q5D7H7), P. pithecia (AAV91986), C. donacophilus (AAV919990), S. sciureus (AAV919888), S. labiatus (AAV91989). (TIF) [file ppat.1002019.s003.tif]

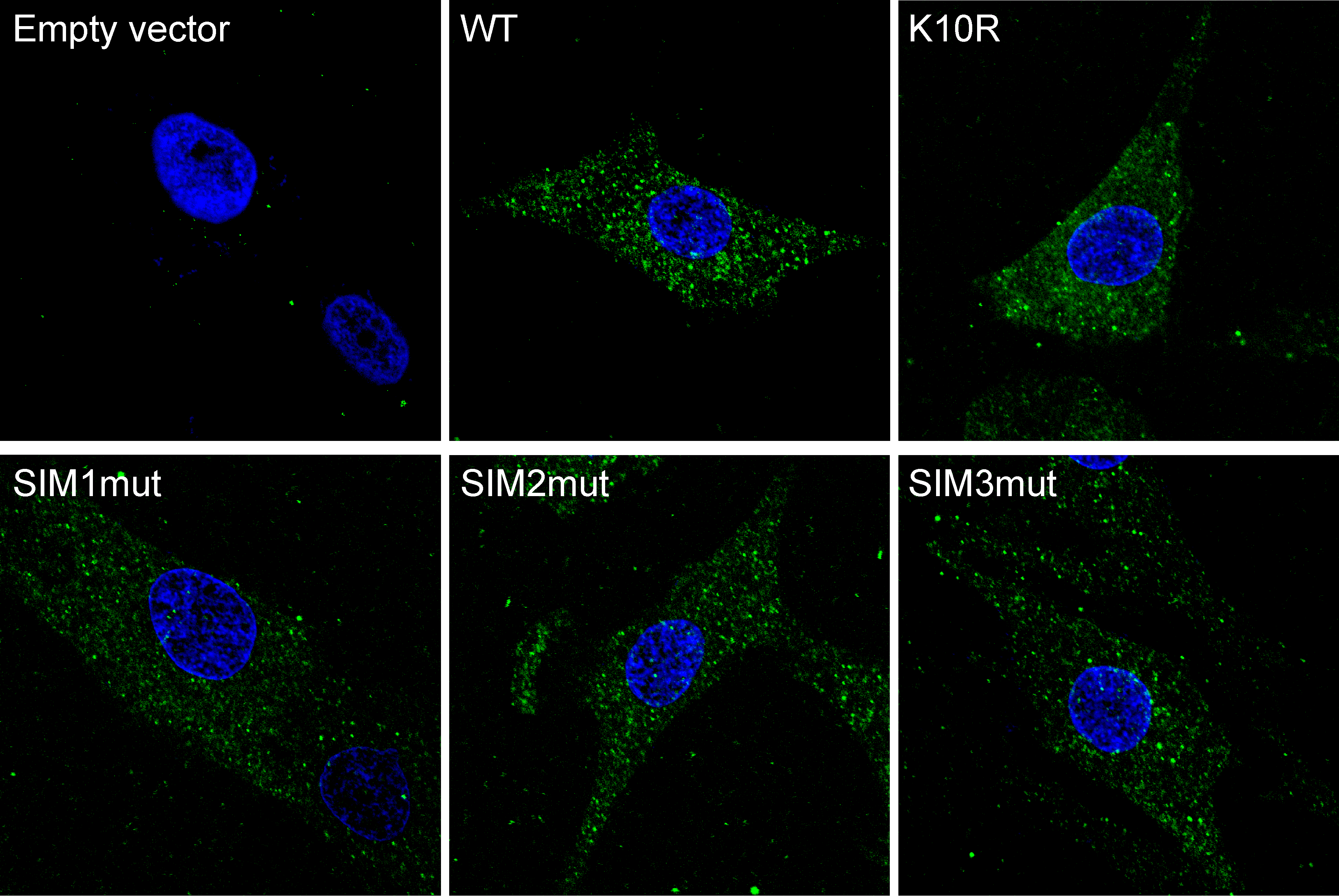

Supplement: Figure S4 — Mutations in TRIM5α do not change its subcellular localization. The subcellular localization of rhesus TRIM5α stably expressed in CRFK cells was assayed. Empty vector control or TRIM5α overexpressing cells were grown on coverslips for twenty-four hours and fixed in 3.7% formaldehyde in PBS. After permeabilization with 1% triton X-100 the cells were incubated with anti-FLAG M2 monoclonal antibody (Sigma) at a dilution of 1∶500 and a secondary anti-mouse conjugated with Alexa Fluor-488 (Molecular probes) at a dilution of 1∶500. Vectashield mounting medium with DAPI (Vector) was used. The cells were visualized with a UPLSAPO 60× 1.35-numerical aperture Olympus oil immersion objective using a Olympus BX61 microscope fitted with a FV1000 FLUO laser scanning confocal system. Image analysis was performed using MethaMorph software. (TIF) [file ppat.1002019.s004.tif]

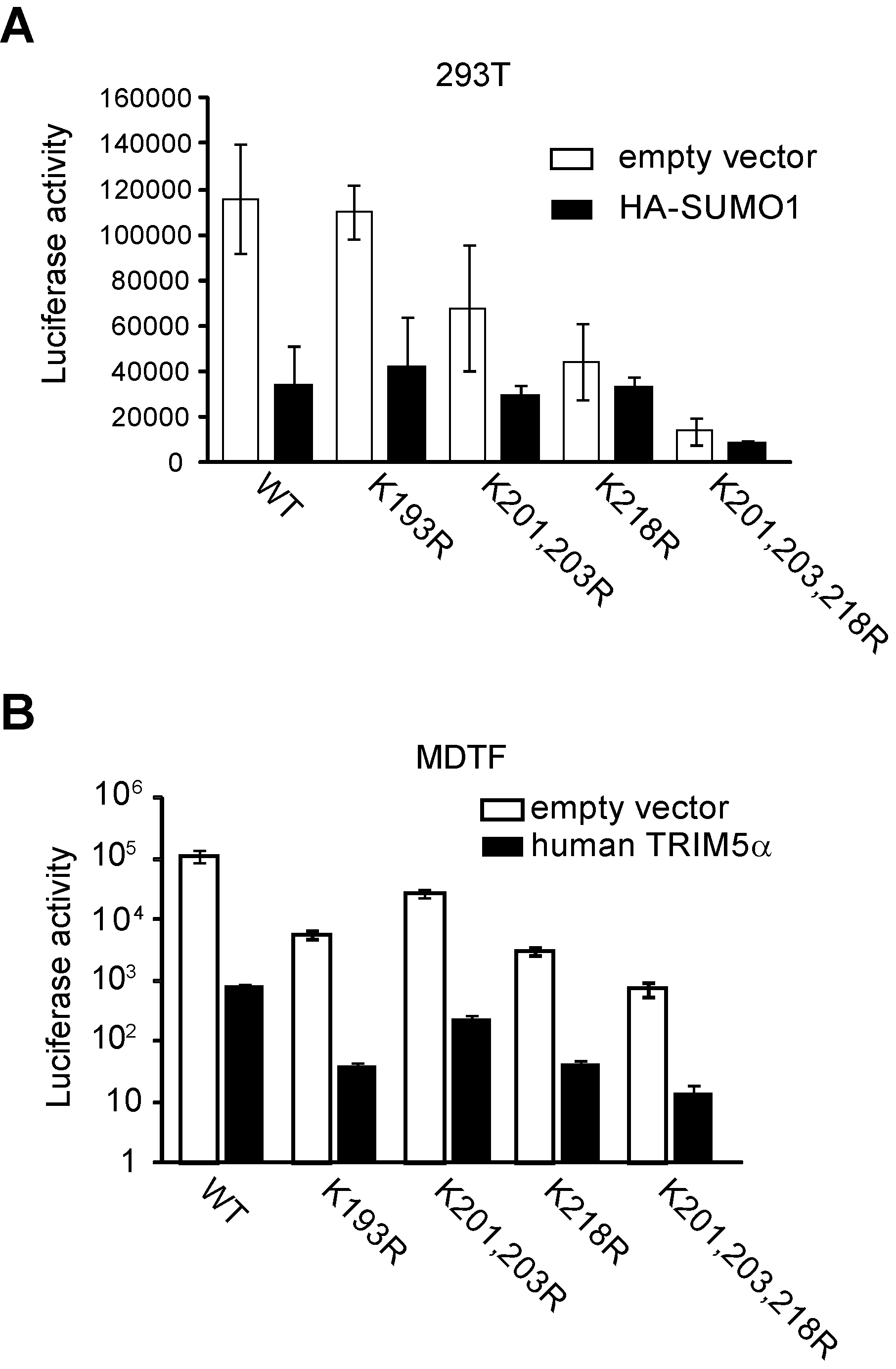

Supplement: Figure S5 — CA mutations altering putative SUMO conjugation site reduce viral infectivity. A. The 293T empty vector and HA-SUMO-1 cell lines were infected with wild type N-MLV luc or N-CA mutant viruses. B. MDTF empty vector and wild-type human TRIM5α cell lines were infected with wild-type N-MLV luc or the N-CA mutant viruses. Forty-eight hours after infection, luciferase activity was measured. One representative of 6 different experiments is shown. Error bars indicate standard deviation of triplicates in the same experiment. (TIF) [file ppat.1002019.s005.tif]
